# There Is Hope After All: Quantifying Opinion and Trustworthiness in Neural Networks

**DOI:** 10.3389/frai.2020.00054

**Published:** 2020-07-31

**Authors:** Mingxi Cheng, Shahin Nazarian, Paul Bogdan

**Affiliations:** Ming Hsieh Department of Electrical and Computer Engineering, University of Southern California, Los Angeles, CA, United States

**Keywords:** artificial intelligence, deep neural networks, machine learning, trust in AI, subjective logic

## Abstract

Artificial Intelligence (AI) plays a fundamental role in the modern world, especially when used as an autonomous decision maker. One common concern nowadays is “how trustworthy the AIs are.” Human operators follow a strict educational curriculum and performance assessment that could be exploited to quantify how much we entrust them. To quantify the trust of AI decision makers, we must go beyond task accuracy especially when facing limited, incomplete, misleading, controversial or noisy datasets. Toward addressing these challenges, we describe DeepTrust, a Subjective Logic (SL) inspired framework that constructs a probabilistic logic description of an AI algorithm and takes into account the trustworthiness of both dataset and inner algorithmic workings. DeepTrust identifies proper multi-layered neural network (NN) topologies that have high projected trust probabilities, even when trained with untrusted data. We show that uncertain opinion of data is not always malicious while evaluating NN's opinion and trustworthiness, whereas the disbelief opinion hurts trust the most. Also trust probability does not necessarily correlate with accuracy. DeepTrust also provides a projected trust probability of NN's prediction, which is useful when the NN generates an over-confident output under problematic datasets. These findings open new analytical avenues for designing and improving the NN topology by optimizing opinion and trustworthiness, along with accuracy, in a multi-objective optimization formulation, subject to space and time constraints.

## 1. Introduction

“AI is no longer the future–it's now here in our living rooms and cars and, often, our pockets” (IBM, 2015). Trust is a significant factor in subjective world, while becoming increasingly critical in Artificial Intelligence (AI). When we behave according to AI's calculated results, how much trust should we put in it? Neural networks (NNs) have been deployed on numerous applications, however, despite their success, such powerful technologies also raise concerns (Rossi, 2019). The incidents, such as fatal accidents of self-driving cars, intensified the concerns on NNs' safety and trustworthiness. Research efforts focused on trustworthiness and safety of NNs include two major aspects: certification and explanation. The former process is held before the arrangement of the model or product to make sure it functions correctly, while the latter tries to explain the behavior of the model or product during its lifetime (Huang et al., 2018). Verification and testing are the two techniques frequently used in certification process, but the explainability of systems with machine learning components is still difficult to achieve for AI developers (Huang et al., 2018). Neural network verification determines whether a property, e.g., safety (Ivanov et al., 2019), local robustness, holds for a neural network. A robust model has the ability to maintain an “acceptable” behavior under exceptional execution conditions (Fernandez et al., 2005), such as adversarial samples (Szegedy et al., 2013). Robustness of NNs has been well-studied in the literature (Huang et al., 2017; Gehr et al., 2018). Trustworthiness of NNs, however, is a more complicated and abstract concept that needs to be explored. In summary, robustness contributes to trustworthiness, but robustness alone is not sufficient for trustworthiness quantification since it only partially covers the verification requirement.

Formal and empirical metrics are designed to model trust. Subjective Logic (SL) is a type of probabilistic logic that explicitly takes uncertainty and source trust into account (Jøsang, 2016). It has been used to model and analyze trust networks in social networks and Bayesian networks (Jøsang et al., 2006), and to evaluate information from untrustworthy sources (Koster et al., 2017). SL offers significantly greater expressiveness than Boolean truth values and probabilities by opinion representation, and gives an analyst the ability to specify vague (and subjective) expressions, such as “I don't know” as input arguments (Jøsang, 2016). Arguments in SL are subjective opinions that contain four parameters: belief, disbelief, uncertainty, and base rate. For example, believing in a statement 100% is an opinion. In SL discipline, this case is expressed as belief is one, disbelief and uncertainty are both zeros. Detailed SL definitions and operators are introduced in section 3. Opinions are also related to the belief representation in Dempster—Shafer belief theory (DST) (Dempster, 2008). We provide a discussion of typical trust modeling and propagation approaches from other disciplines in section 2. Compared to monotonic logics, SL provides multiple advantages while handling default reasoning, abductive reasoning, and belief reasoning. The detailed comparison is discussed in section 2. Uncertainty quantification is closely related to trust evaluation. Various works in the scientific literature have explored uncertainty in deep learning and machine learning models. In comparison to uncertainty propagation (Titensky et al., 2018), the term uncertainty in this work refers to the uncertainty value in a subjective opinion of human or machine observer. A detailed prior uncertainty work review can be found in section 2.

In this work, we propose DeepTrust, an SL-inspired framework to evaluate the opinion and trustworthiness of an AI agent, such as a neural network, and its predictions, based on input trust information, a hyper parameter of neural networks (i.e., topology), and parameters of neural networks (such as weight and bias). The questions about “how much we should trust AI and input data” and “Which topologies are more trustworthy” can be answered by evaluating AI in the SL discipline offered by DeepTrust. The trust quantification in this work is not limited by the linearity and/or non-linearity of the neural network and the size or topology of the neural network. By providing the projected trust probability, DeepTrust promises the needed trustworthiness in a wide range of applications involving problematic data. One example is related to the 2016 presidential election prediction. We show that differently from almost all the predictors back in 2016, DeepTrust calculates a very low projected trust probability of 0.21 for “Clinton wins,” and a higher projected trust probability of 0.5 for “Trump wins.” Hence, by quantifying the opinion and trustworthiness, DeepTrust could relatively predict that it would be more than twice as trustworthy to predict Trump as the winner, or at least it could raise alarms on all those strong pre-election results in favor of Clinton.

The contributions of this work are stated as follows:

- We define the trustworthiness and propose a framework to quantify the trustworthiness of a multi-layered neural network based on SL.
- We confirmed that the untrustworthy data causes a decrease of neural network trustworthiness. Different neural network topologies react very differently when facing with untrustworthy data. A good topology leads to relatively high trustworthiness, even when the training data is untrustworthy. In addition, the accuracy and trustworthiness of a neural network are not necessarily correlated.
- We verified that uncertainty is not always malicious when evaluating the trustworthiness of a NN. In the case of maximum data uncertainty, there is a hope of belief in the neural network trained with such data. Compared to the uncertainty, the disbelief in data hurts the trustworthiness the most.

This paper is organized as follows. Section 2 introduces the prior works in trust modeling from other disciplines and uncertainty quantification in AI and deep learning, as well as a discussion on monotonic logics. We provide an introduction of SL in section 3, to offer the necessary background knowledge needed for understanding the proposed DeepTrust along with intuitive examples. The proposed methods of opinion and trustworthiness quantification of NNs and NNs' predictions are introduced in section 4, where we also provide the definition of trustworthiness. Note that the NNs in this work are multi-layered NNs with more than one hidden layers between the input and output layer and without convolutional and recurrent neurons. The two major findings and minor remarks along with the experimental results are presented in section 5. Discussion is given in section 6. Finally, the related SL information and examples and additional experimental results are provided in Supplementary Materials.

## 2. Prior Works

To measure the degree of trust, several formal metrics are designed to model and reason about trust. SL is one of the formal metrics that combines probability distribution with uncertainty, and the opinion is a distribution of probability distributions. An opinion in SL can be expressed as a quadruplet {*belief, disbelief, uncertainty, base rate*}, which embeds the uncertainty into a computation process explicitly. Belief, disbelief, and uncertainty are dependent and have to add up to 1. The subjective opinions express uncertainty and vagueness of crisp domains via belief, disbelief, and uncertainty. Use “height of a person” as an example, the crisp domains in SL consist of terms, such as 180 cm, the opinion of this value could be 0.5 belief and 0.5 uncertainty. On a related note, fuzzy logic (Novák et al., 2012) is another formal trust metric where the domains for variables consist of terms with uncertainty and vagueness (Jøsang, 2016). In the case of “height of a person,” the possible values in fuzzy logic could be “short,” “average,” or “tall.” A person measuring 180 cm could be considered 0.5 tall and 0.5 average. Therefore, SL and fuzzy logic handle different aspects of uncertainty and vagueness. The combination of these two reasoning frameworks needs to be explored in the future.

Reasoning with uncertainty based on evidence has been developed since 1960s. The theory of belief functions, DST, also referred to as evidence theory, is developed for modeling epistemic uncertainty-a mathematical theory of evidence (Shafer, 1976; Dempster, 2008). Many authors have later proposed different rules for combining evidence, often with a view of better handling conflict evidence (Sentz and Ferson, 2002), such as SL. DST is highly expressive by including uncertainty about the probabilities. It has advantages over probabilities because the use of beliefs enables DST to express “I don't know” as an input to a reasoning model. The belief mass distribution in DST is called a basic belief assignment, which is equivalent to the belief/uncertainty representation of SL (Jøsang, 2016). However, there has been considerable confusion and controversy around the adequacy of belief fusion operators in DST (Smarandache, 2004). SL contains the corresponding operator in DST (the belief constraint fusion operator) and has more fusion operators that are appropriate for different situations [e.g., cumulative belief fusion operator is used when the amount of independent evidence increases by including more source. Also average belief fusion operator is used when dependence between sources is assumed (Jøsang, 2016)]. The fusion operator we utilize in DeepTrust is the averaging fusion in SL, which is introduced in section 3.4. On a related note, trust measuring is closely related to reputation systems that allow users to build trust through reputation. Trust propagation models in such social network systems or online reputation systems are well-studied in the literature (Guha et al., 2004; Su et al., 2014; Urena et al., 2019). However, these trust propagation approaches haven't been applied and utilized in AI disciplines. Spreading activation models (Quillan, 1966) have played important roles in trust propagation and reputation systems (Kovacs and Ueno, 2006; Wang et al., 2007; Troussov et al., 2009). They were proposed initially to simulate human comprehension in semantic networks (Ziegler and Lausen, 2004). Spreading factor is a crucial variable in spreading activation model-based trust propagation networks. It is a real number ranging from zero to one. Intuitively, spreading factor is the ratio between direct trust in a node *x* in some trust network and trust in the ability of *x* to recommend others as trustworthy peers (Ziegler and Lausen, 2005). The concepts in spreading activation models have a lot of similarities with SL, such as trust networks and trust propagation. However, spreading activation models are less comprehensive and expressive, and haven't been applied to trust quantification in neural networks.

Compared to monotonic logics which indicate that learning a new piece of knowledge cannot reduce the set of what is known, non-monotonic logics (NMLs) whose consequence relations are not monotonic and are devised to capture and represent defeasible inference (Strasser and Antonelli, 2019). Among many NMLs research results in the literature we recall the McCarthy's circumscription (McCarthy, 1980), the defeasible reasoning (Strasser and Antonelli, 2019), the default reasoning (Reiter, 1980; Antoniou, 1999; Friedman et al., 2000), the autoepistemic logic (Moore, 1984; Konolige, 1987), the stable model semantics (Gelfond and Lifschitz, 1988) that closely related to autoepistemic logic and default logic, the abductive reasoning (Josephson and Josephson, 1996; Aliseda, 2017), the causal reasoning (Falcon, 2019), and the belief revision (Goldszmidt and Pearl, 1992; Williams, 1994). SL embeds factors that can help handling default reasoning, abductive reasoning, belief revision, and intercausal reasoning. Default logic is a NML that can express facts like “by default, something is true”; by contrast, standard logic can only express that something is true or false. In SL, the concept of base rates represents exactly “by default, something is true,” which is central in the theory of probability. For example, base rates are needed for default reasoning, for Bayes' theorem, for abductive reasoning and for Bayesian updating. Similarly to defeasible inference, abductive reasoning allows for a retraction of inference (Strasser and Antonelli, 2019). Abductive reasoning is a form of logical inference which starts with an observation or set of observations and then tries to find the simplest and most likely explanation for the observations. In SL, conditional reasoning is proposed to reason from belief about consequent propositions to infer belief about antecedent propositions, which commonly is called abductive reasoning. In addition, subjective abduction (Jøsang, 2016) states how SL can be used for reasoning in the opposite directions to that of the conditionals, which typically involves the subjective version of Bayes' theorem. Belief revision is the process of changing beliefs to take into account a new piece of information. In SL, a corresponding method is called trust revision, which is needed for dealing with the conflict opinions. A simplistic strategy is to fuse the conflicting opinions. Causal reasoning is the process of identifying causality (Falcon, 2019). SL supports subjective intercausal reasoning, which takes place in two steps: abduction and division. Therefore, compared to monotonic logic, SL is able to handle many cases that cannot be solved in monotonic logics and the advantages of the afore-mentioned NML properties therefore motivate the utilization of SL.

Nowadays, the need for uncertainty quantification in machine-assisted decision-making is rising, especially when human's security and safety are at stake (Begoli et al., 2019). When applying feedforward neural networks to reinforcement learning or supervised learning, uncertainty in the training data usually cannot be assessed, hence overly confident decisions or predictions are typically made based on the fully trusted data (Blundell et al., 2015). Motivated by overfitting, Geifman et al. (2019) estimate uncertainty of highly confident points by utilizing earlier snapshots of the trained model, i.e., before the estimation shakes. Uncertainty in Geifman's work is defined as negative confidence, which is totally different than what we quantify in DeepTrust via SL. Negative confidence fundamentally measures a similar quantity as confidence, and confidence is the output provided along with the accuracy. Gal and Ghahramani (2016) build a probabilistic interpretation of dropout to obtain model uncertainty, i.e., epistemic uncertainty, out of existing deep learning models. Oh et al. (2019) propose a model to measure input uncertainty (aleatoric uncertainty) by “hedging” the location of each input in the embedding space. In these approaches, uncertainty is defined differently, such as negative of confidence and first-order uncertainty, while uncertainty in DeepTrust is second-order, as defined in SL. Traditional probability represents first-order uncertainty (Sundgren and Karlsson, 2013). Second-order uncertainty is represented in terms of a probability density function over first-order probabilities. In uncertainty propagation, a pre-trained neural network accepts an input and generates output, and uncertainty from input propagates through the NN resulting in uncertainty of the output (Titensky et al., 2018). Research efforts concentrating on uncertainty measure in NNs conduct uncertainty propagation through the entire neural network, such as Monte Carlo sampling, unscented transform, or layer by layer, such as piece-wise exponential approximation (Astudillo and Neto, 2011). Sensoy et al. (2018) interpret the behavior of predictor differently from an evidential reasoning perspective and build the link from predictions to the belief and uncertainty of SL. In contrast, DeepTrust considers the topologies of multi-layered NNs and utilizes evidential reasoning in a different way as described in section 4.

The first order uncertainties defined in afore-mentioned works are suitable for their defined circumstances. An important uncertainty quantification direction in the scientific literature is the distinction between aleatory and epistemic uncertainty, both of which are first order uncertainties. Aleatory uncertainty is the same as statistical uncertainty, as it expresses that for a repeated realization of an experiment, we only know the long-term relative frequency of the outcomes, but we do not know the outcome of each run of the experiment. A simple example is flipping a coin. Epistemic uncertainty is the same as systematic uncertainty with focusing attention on a single experiment. It expresses that we could in principle know the outcome of a specific event, but we do not have enough evidence to know it exactly. For example, the assassination of President Kennedy is believed to have been committed by Lee Harvey Oswald, but there is considerable uncertainty around it (Jøsang, 2016). This event only happened once, so the long-term relative frequency does not make sense. Aleatory and epistemic uncertainties of a binary variable are greatest when the probability of this variable is 50%. However, when an opinion has projected trust probability (which is introduced in section 3.2) of 50%, it says nothing about the exact value of the uncertainty mass. The uncertainty mass could range from a minimum value of zero to a maximum value of one. In this case, the explicit value of second order uncertainty provides some information which makes it richer than the first order uncertainties. Similarly, second-order uncertainty is preferred in some neuroscience works, such as in Bach et al. (2011) since factors other than second-order uncertainty may confound experimental manipulations of ambiguity. An opinion in SL can contain uncertainty mass in the sense of uncertainty about probabilities. Interpreting uncertainty mass as vacuity of evidence reflects the property that “the fewer observations the more the uncertainty mass.” The mapping between binomial opinion and Beta PDF (introduced in section 3.2) provides the definition of uncertainty mass, which is based on the evidence.

## 3. Background Knowledge of Subjective Logic

This section briefly introduces the related background knowledge of SL. Differently from binary logic and operators which are based on true/false logic values and seem more familiar, SL defines its own set of logic and operators, not only based on logic truth, but also based on probabilistic uncertainty. In the following we introduce the basic definitions and operators that are used in DeepTrust, specifically, binomial opinions and their quantification from evidence, the binomial multiplication operator, and averaging fusion operator.

### 3.1. Binomial Opinions

We start with an example to explain what opinions mean in the real world. Imagine you would like to purchase a product from a website, and you have an opinion about this product, i.e., belief in support of the product being good, belief in support of the product not being good (or disbelief in support of the product being good), uncertainty about the product, and prior probability of the product being good. A *binomial opinion* about the truth of *x*, e.g., a product, is the ordered quadruplet {*belief, disbelief, uncertainty, base rate*}, which is denoted by ${\bar{W}}_{x}=\left\{b_{x},d_{x},u_{x},a_{x}\right\}$, with an additional requirement: *b*_*x*_ + *d*_*x*_ + *u*_*x*_ = 1, where *b*_*x*_, *d*_*x*_, *u*_*x*_ ∈ [0, 1]. The respective parameters are: *belief mass* *b*_*x*_, *disbelief mass* *d*_*x*_, *uncertainty mass* *u*_*x*_, that represents the vacuity of evidence, and *base rate* *a*_*x*_, the prior probability of *x* without any evidence. In what follows we will use the *binomial opinion* and *opinion* interchangeably.

### 3.2. Binomial Opinion Quantification From Evidence

Let ${\bar{W}}_{x}=\left\{b_{x},d_{x},u_{x},a_{x}\right\}$ be a binomial opinion of a random variable *X*, e.g., a product. To formulate our opinion about this product, we need to rely on the evidences about the quality of this product. To calculate the *binomial opinion* of random variable *X* from directly observed evidence, we use the following mapping rule (Jøsang, 2016; Cho and Adali, 2018):

$$\{\begin{array}{l} b_{x}=\frac{r_{x}}{r_{x}+s_{x}+W},\\ d_{x}=\frac{s_{x}}{r_{x}+s_{x}+W},\\ u_{x}=\frac{W}{r_{x}+s_{x}+W}. \end{array}$$

*r*_*x*_ and *s*_*x*_ represent the positive evidence and negative evidence of *X* taking value *x*, respectively. *W* is a non-informative prior weight, which has a default value of 2 to ensure that the prior probability distribution function (PDF) is the uniform PDF when *r*_*x*_ = *s*_*x*_ = 0 and *a*_*x*_ = 0.5 (Jøsang, 2016). In our online shopping example, a customer can form his/her opinion by looking at the product report. For example, 10 reports show that the product is good and 10 show that it is bad. Then positive evidence *r*_*x*_ = 10 and negative evidence *s*_*x*_ = 10, and the opinion can be calculated based on these evidence (and say a *W* of 2) as ${\bar{W}}_{x}=\left\{\frac{10}{22},\frac{10}{22},\frac{2}{22},\frac{1}{2}\right\}$.

To further understand the binomial opinion, we introduce the projected probability *p*_*x*_ = *b*_*x*_ + *u*_*x*_ * *a*_*x*_. A binomial opinion is equivalent to a Beta probability density function (PDF). Assume a random variable *X* is drawn from a binary domain $\left\{x,\bar{x}\right\}$. Let *p* denote a continuous probability function *p*:*X* → [0, 1] where $p\left(x\right)+p\left(\bar{x}\right)=1$. With *p*(*x*) as variable, the Beta probability density function *Beta*(*p*(*x*), α, β) reads:

$$\begin{array}{l} Beta\left(p\left(x\right),\alpha ,\beta \right)=\frac{\Gamma \left(\alpha +\beta \right)}{\Gamma \left(\alpha \right)\Gamma \left(\beta \right)}{\left(p\left(x\right)\right)}^{\alpha -1}{\left(1-p\left(x\right)\right)}^{\beta -1},\\ \;\alpha >0,\beta >0, \end{array}$$

where α, β represent evidence/observations of *X* = *x* and $X=\bar{x}$, respectively. Let *r*_*x*_ and *s*_*x*_ denote the number of observations of *x* and $\bar{x}$, then α, β can be expressed as follows:

$$\{\begin{array}{l} \alpha =r_{x}+a_{x}W,\\ \beta =s_{x}+(1-a_{x})W. \end{array}$$

The bijective mapping between a binomial opinion and a Beta PDF emerges from the intuitive requirement that the projected probability of a binomial opinion must be equal to the expected probability of a Beta PDF, i.e., $p_{x}=b_{x}+u_{x}*a_{x}=E\left[x\right]=\frac{\alpha }{\alpha +\beta }=\frac{r_{x}+a_{x}W}{r_{x}+s_{x}+W}$.

### 3.3. Binomial Multiplication

Binomial multiplication operator is used to derive the opinion of the conjunction of two opinions. Multiplication in SL corresponds to AND in binary logic. Let ${\bar{W}}_{x}=\left\{b_{x},d_{x},u_{x},a_{x}\right\}$ and ${\bar{W}}_{y}=\left\{b_{y},d_{y},u_{y},a_{y}\right\}$ be binomial opinions about *x* and *y*, respectively. We can get the opinion of the conjunction *x* ∧ *y* (Jøsang, 2016):

$${\bar{W}}_{x\cdot y}={\bar{W}}_{x}\cdot {\bar{W}}_{y}:\{\begin{array}{l} b_{x\wedge y}=b_{x}b_{y}+\frac{(1-a_{x})a_{y}b_{x}u_{y}+a_{x}(1-a_{y})b_{y}u_{x}}{1-a_{x}a_{y}},\\ d_{x\wedge y}=d_{x}+d_{y}-d_{x}d_{y},\\ u_{x\wedge y}=u_{x}u_{y}+\frac{(1-a_{y})b_{x}u_{y}+(1-a_{x})b_{y}u_{x}}{1-a_{x}a_{y}},\\ a_{x\wedge y}=a_{x}a_{y}. \end{array}$$

In our online shopping example, assume a customer holds opinions of two different products *x* and *y*. Then he/she can derive the opinion about the conjunction *x* ∧ *y* using this multiplication operator.

### 3.4. Averaging Fusion

To combine different people's opinions about the same domain, we use *fusion* operators. The fusion operator used in this work is the *averaging fusion* operator (Wang and Zhang, 2017), which is appropriate for circumstances when agent *A* and agent *B* observe the same process over that same time period (Jøsang, 2016). In what follows we will use *fusion* and *averaging fusion* interchangeably. Let ${\bar{W}}_{X}^{A}=\left\{b_{X}^{A},d_{X}^{A},u_{X}^{A},a_{X}^{A}\right\}$ and ${\bar{W}}_{X}^{B}=\left\{b_{X}^{B},d_{X}^{B},u_{X}^{B},a_{X}^{B}\right\}$ be source agent *A* and *B*'s binomial opinions about *X*, respectively. For example, customer *A* and *B*'s opinions about a product. The binomial opinion ${\bar{W}}_{X}^{\left(A◇B\right)}=fusion\left({\bar{W}}_{X}^{A},{\bar{W}}_{X}^{B}\right)$ is called the averaged opinion of ${\bar{W}}_{X}^{A}$ and ${\bar{W}}_{X}^{B}$, which represents the combined opinion of product *X* from customer *A* and *B*. The averaging fusion operator works as follows:

Case I: $u_{X}^{A}\neq 0$ or $u_{X}^{B}\neq 0$.

$${\bar{W}}_{X}^{\left(A⋄B\right)}:\{\begin{array}{l} b_{X}^{\left(A⋄B\right)}=\frac{b_{X}^{A}u_{X}^{B}+b_{X}^{B}u_{X}^{A}}{u_{X}^{A}+u_{X}^{B}},\\ u_{X}^{\left(A⋄B\right)}=\frac{2u_{X}^{A}u_{X}^{B}}{u_{X}^{A}+u_{X}^{B}},\\ a_{X}^{\left(A⋄B\right)}=\frac{a_{X}^{A}+a_{X}^{B}}{2}. \end{array}$$

Case II: $u_{X}^{A}=0$ and $u_{X}^{B}=0$:

$${\bar{W}}_{X}^{\left(A⋄B\right)}:\{\begin{array}{l} b_{X}^{\left(A⋄B\right)}=\gamma_{X}^{A}b_{X}^{A}+\gamma_{X}^{B}b_{X}^{B},\\ u_{X}^{\left(A⋄B\right)}=0,\\ a_{X}^{\left(A⋄B\right)}=\gamma_{X}^{A}a_{X}^{A}+\gamma_{X}^{B}a_{X}^{B}, \end{array}$$

where

$$\{\begin{array}{l} \gamma_{X}^{A}=lim_{u_{X}^{A}\to 0,u_{X}^{B}\to 0}\frac{u_{X}^{B}}{u_{X}^{A}+u_{X}^{B}},\\ \gamma_{X}^{B}=lim_{u_{X}^{A}\to 0,u_{X}^{B}\to 0}\frac{u_{X}^{A}}{u_{X}^{A}+u_{X}^{B}}. \end{array}$$

## 4. Methods

We define trustworthiness and introduce DeepTrust, our neural network opinion and trustworthiness quantification framework in this section. We evaluate the opinion in a simple one neuron case, then further generalize to multi-layered typologies.

### 4.1. Trustworthiness

It is crucial in any stochastic decision-making problems to know whether the information about the probabilistic description is trustworthy and if so, the degree of *trustworthiness*. This is even more important when quantifying trust in an artificial intelligence-based decision-making problem. In scenarios without trust quantification, a neural network can only present the decision based on output labels without any clear measure of trust in those decisions. However, if the environment is corrupted and the network is trained with damaged data, the decisions generated by this network could be highly untrustworthy, e.g., YES but with 10% belief. Lack of quantification of trust, results in lack of information, and consequently fatal errors. This shows the need for trust quantification. In what follows, we describe DeepTrust for quantifying the opinion and trustworthiness of a multi-layered NN as a function of its topology and the opinion about the training datasets. DeepTrust applies to both classification and regression problems since the value of input does not affect the calculation of the opinion. As long as we have true labels, i.e., the problem is in the realm of supervised learning, DeepTrust can calculate the trustworthiness.

**Definition 4.1**. In our DeepTrust, we define the *trustworthiness* of *x* as the *projected trust probability* of *x*, i.e., trustworthiness *p*_*x*_ = *b*_*x*_ + *u*_*x*_ * *a*_*x*_, where *b*_*x*_, *u*_*x*_, *a*_*x*_ are belief, uncertainty, and base rate of *x*, respectively.

The intuition for our definition of trustworthiness is as follows. The higher the belief mass and the base rate are, the higher the projected trust probability and hence the higher the trustworthiness is. If a neural network results in high projected trust probability, then it is considered to be trustworthy. Belief mass, product of uncertainty mass and base rate can both contribute to projected trust probability. High belief mass comes from a large volume of positive evidence supporting *x* and high base rate represents high prior probability of *x* without any evidence. For example, when a large volume of evidence is collected, if *b*_*x*_ = 0, i.e., belief is zero, then it can be concluded that all collected evidence are not supporting *x*, hence *d*_*x*_ = 1, i.e., disbelief is one. Now the trustworthiness of *x* should be extremely low since no evidence is supporting it, and *p*_*x*_ = 0. An opposite case is when no evidence supports or opposes *x*, the background information about *x*, i.e., *a*_*x*_, defines the trustworthiness of *x* due to the lack of evidence. In this case, *b*_*x*_ = *d*_*x*_ = 0, i.e., belief and disbelief both equal to zero, uncertainty *u*_*x*_ = 1, and *p*_*x*_ = *a*_*x*_, i.e., projected probability equals to base rate. It is noteworthy that our measure of trust based on projected trust probability is in agreement with the main SL reference (Jøsang, 2016). More precisely Josang presents the special case of projected trust probability as 1 (0), as a case with complete trust (distrust) in the source.

### 4.2. DeepTrust Formulation

**Figure 1:**
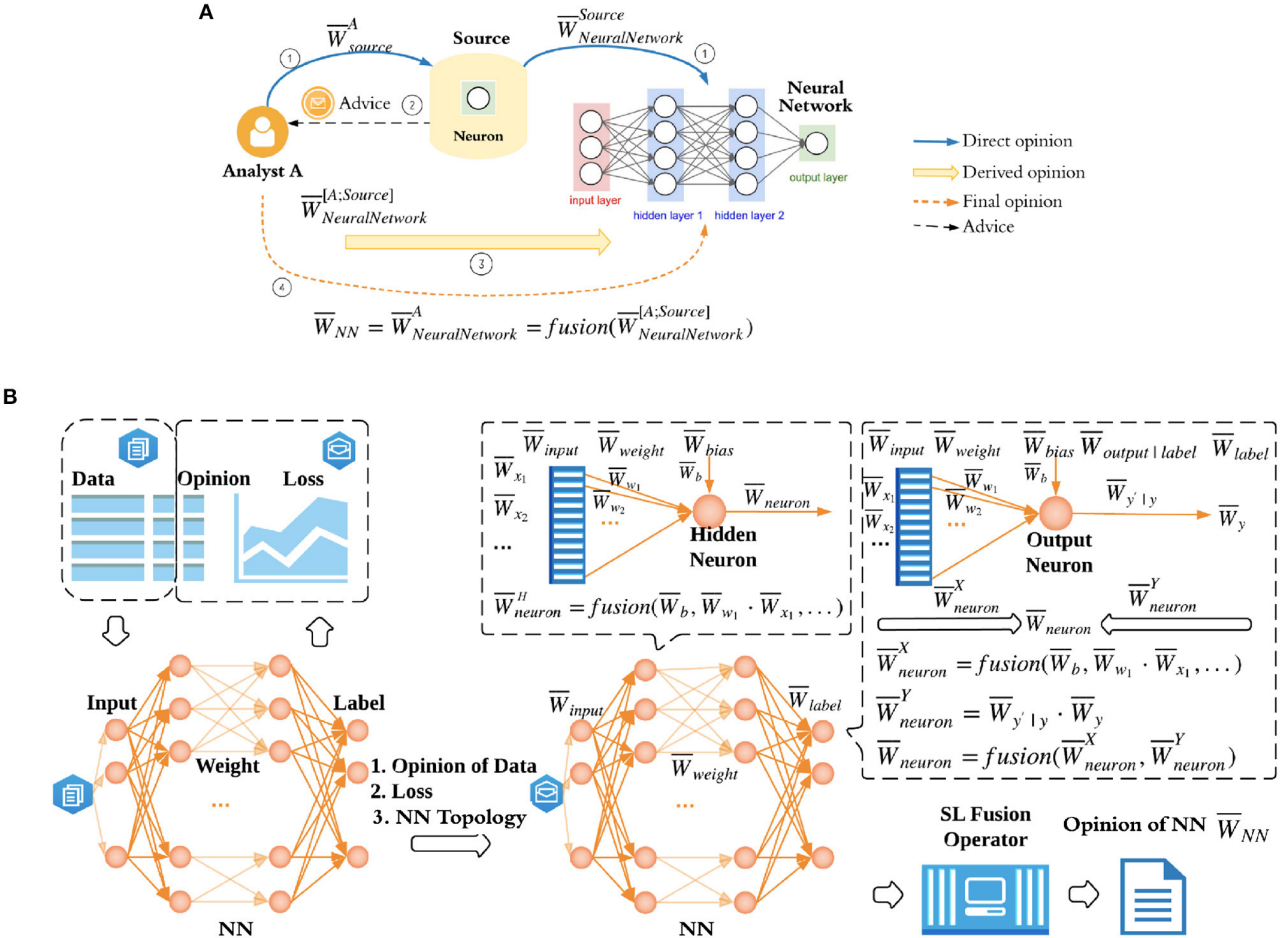
DeepTrust: subjective trust network formulation for multi-layered NNs and NN opinion evaluation. **(A)** Subjective trust network formulation for a multi-layered NN. To quantify the opinion of network ${\bar{W}}_{NN}$, i.e., human observer's opinion of a particular neural network ${\bar{W}}_{NeuralNetwork}^{A}$, human observer as an analyst relies on sources, in this case neurons in network, which hold direct opinions of neuron network. ${\bar{W}}_{source}^{A}$ and ${\bar{W}}_{NeuralNetwork}^{source}$ are analyst A's opinion of source and source's opinion of neural network. Then the derived opinion of neural network is calculated as $fusion\left({\bar{W}}_{NeuralNetwork}^{\left[A;source\right]}\right)$. **(B)** NN opinion evaluation. Dataset in DeepTrust contains *Data*, i.e., features and labels the same way as a normal dataset, in addition to the *Opinion* on each data point. If a data point doesn't convey the information as other data points do, for example, one of the features is noisy or the label is vague, we consider this data as uncertain, and hence introduce uncertainty into dataset. Rather than assigning different opinions to each feature and label, we assign a single opinion to the whole data point. The reason is described in section 4.3. Given NN topology, opinion of data, and training loss, DeepTrust can calculate the trust of NN. Note that, the trust of hidden neurons and trust of output neurons are quantified differently as shown in this figure. Each neuron in output layer is a source which provides advice to analyst, so that the analyst can derive its own opinion of the NN. ${\bar{W}}_{neuron|y}^{Y}$ is represented by ${\bar{W}}_{y^{\prime }|y}$ for simplicity. Detailed computation and explanation are summarized in Section 4. After trust of all output neurons are evaluated, SL fusion operator described in Section 3.4 is used to generate final opinion of neural network ${\bar{W}}_{NN}$.

Because we cannot apply SL directly to trust quantification of NNs, we will need to formulate this as a SL problem. For that we formulate the trust relationships in NN trust quantification as a *subjective trust network* as shown in Figure 1A, where NN is the target object. A subjective trust network represents trust and belief relationships from agents, via other agents or sources to target entities/variables, where each trust and belief relationship is expressed as an opinion (Jøsang, 2016). For example, a customer wants to purchase a product from a website, so he/she will make a purchase decision by browsing other buyers' reviews of this product. He/she may or may not trust each and every review. In this case, the customer as an agent forms his/her opinion of the product via other agents' (i.e., buyers') opinions. More details about subjective trust network is described in Supplementary Material Section 2.

In DeepTrust, a human observer A as an analyst wants to formulate an opinion about a given neural network, ${\bar{W}}_{NeuralNetwork}^{A}$. However, this analyst A doesn't have the direct trust relationship with the whole neural network and hence needs to gather sources' opinions of the neural network, ${\bar{W}}_{NeuralNetwork}^{Source}$'s. In the case of neural networks, the sources are the neurons in the output layer. Therefore, the human observer can only interpret the trustworthiness of a neural network by referring to the neurons in the output layer. We will later prove in Theorem 4.1 that taking neurons in all layers into consideration causes an opinion duplication. In the trust network in Figure 1A, analyst A discounts the information provided by the source (neuron) and derives an opinion about the neural network, ${\bar{W}}_{NeuralNetwork}^{\left[A;Source\right]}$, i.e., A's opinion of the neural network through a source. An intuitive underlying relationship is that A trusts the source, and the source trusts the neural network. Analyst A discounts the information given by the source since A may not fully trust the source. If there are more than one sources, i.e., more than one neurons in output layer, analyst A will gather advice from all sources and fuse the discounted opinions by fusion operator we introduced in section 3.4. A's opinion to source ${\bar{W}}_{source}^{A}$ is set to be maximum belief based on the assumption that analyst A trusts the source completely, so A doesn't discount the source's information and the derived opinion ${\bar{W}}_{NeuralNetwork}^{\left[A;Source\right]}$ simply becomes ${\bar{W}}_{NeuralNetwork}^{Source}$. Therefore, A's opinion of a given neural network with multiple sources reads:

(1) $$\begin{array}{l} {\bar{W}}_{NeuralNetwork}^{A}=fusion\left({\bar{W}}_{NeuralNetwork}^{\left[A;Source\right]}\right)=fusion\left({\bar{W}}_{NeuralNetwork}^{Source}\right). \end{array}$$

Since neurons in output layer are analyst's sources, we use notation ${\bar{W}}_{neuron}$ in Figure 1B to represent a neuron's opinion ${\bar{W}}_{NeuralNetwork}^{Source}$ in output layer for simplicity, and use notation ${\bar{W}}_{neuron}^{H}$ to represent neurons in hidden layers. Derivations of ${\bar{W}}_{neuron}$ and ${\bar{W}}_{neuron}^{H}$ are introduced in section 4.3 and 4.4. We will omit notation A and denote opinion of neural network as ${\bar{W}}_{NN}$ from now on. As shown in Figure 1B, DeepTrust quantifies NN's opinion based on opinion of dataset, network topology, and training loss. Opinion of dataset is assumed given in this work since it should be quantified and provided by the data collector before the delivery of data (and dataset's opinion and trustworthiness quantification will be explored in future work). We consider multilayer neural networks in this work, and more complicated neural networks, such as convolutional neural networks and recurrent neural networks are left out to be discussed in a future work.

### 4.3. Opinion Evaluation for One Neuron

To better understand the opinion and trustworthiness evaluation for a multi-layered NN, we will first introduce opinion quantification for one neuron with one input and one output. This is later utilized as a foundation of multi-layered NN opinion and trustworthiness quantification by DeepTrust. To calculate the trustworthiness of a NN, we first need the opinions of the data. ${\bar{W}}_{X}$, the opinion of input *X* and ${\bar{W}}_{Y}$, the opinion of true label *Y* are given along with the dataset and used to evaluate the opinion of one neuron *N*. In this work, without losing generality, we assume opinions of all data points ${\bar{W}}_{\vec{x}}$'s and ${\bar{W}}_{y}$'s are the same. The reason is that for realistic datasets, if a data point is damaged or noisy, we may not be able to determine which feature(s) or label is problematic. We would like to note that the size of $\vec{x}$ is greater and equal than 1 in general, and we take it as 1 here since here we are considering a neuron with only one input.

**Figure 2:**
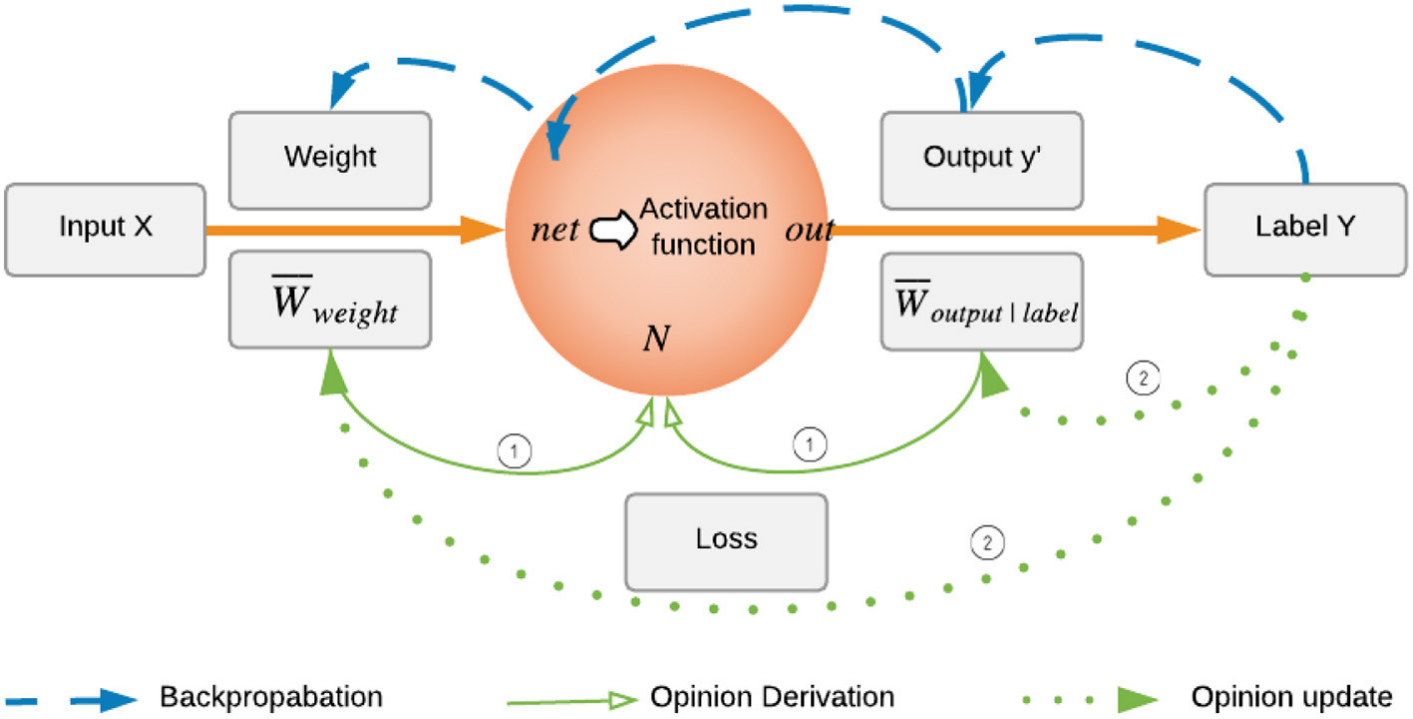
Backpropagation in one neuron and opinion update of weight and output. The backpropagation process in neural network training first compares the true label and output given by the neuron, then back propagates the difference to *net*, and adjusts the weight accordingly to minimize the error. The weight opinion update process mimics the backpropagation: (i) At current episode, the opinion of neuron is the combined opinion of forward opinion and backward opinion, which are based on current ${\bar{W}}_{weight}$, and current ${\bar{W}}_{output|label}$, respectively. (ii) Then in the next episode, the opinion of neuron will be recalculated by taking updated ${\bar{W}}_{weight}$ and ${\bar{W}}_{output|label}$ into consideration.

We first take a look at how the neuron works in an ordinary neural network. When training a neural network, the forward pass takes input, weight, and bias to calculate the total net input *net* as *weight*·*input* + *bias* for hidden neuron *N*. Activation function, such as ReLU is applied to *net* to calculate output *out* of neuron *N*, which is denoted by *y*′ in one neuron case. In backpropagation, the back pass takes error and back propagate it all the way back to the input layer as shown in Figure 2. Inspired by this flow, the opinion of neuron, ${\bar{W}}_{N}$, is calculated based on the forward pass and backward pass operations using two opinions: forward opinion of neuron ${\bar{W}}_{N}^{X}$ from *X* point of view, and backward opinion of neuron ${\bar{W}}_{N}^{Y}$ from *Y* point of view. We can view ${\bar{W}}_{N}^{X}$ and ${\bar{W}}_{N}^{Y}$ as advice from sources *X* and *Y*, respectively. To combine these two opinions, the fusion operator is then used to get the final opinion of neuron ${\bar{W}}_{N}$:

(2) $$\begin{array}{l} {\bar{W}}_{N}=fusion\left({\bar{W}}_{N}^{X},{\bar{W}}_{N}^{Y}\right). \end{array}$$

Here in the one neuron case, *N* is the only neuron in hidden and output layer, hence afore-mentioned ${\bar{W}}_{neuron}$ and ${\bar{W}}_{neuron}^{H}$ are the same and calculated as ${\bar{W}}_{N}$, since this neuron is in hidden/output layer.

To calculate ${\bar{W}}_{N}^{X}$ in Equation (2), we first look at the original neural network and the calculation of net input: $net=\vec{w}\cdot \vec{x}+b$, where weight $\vec{w}$ and bias *b* are initialized randomly. Inspired by this, the forward opinion of *N*, ${\bar{W}}_{N}^{X}$, is calculated as follows:

(3) $$\begin{array}{l} {\bar{W}}_{N}^{X}=fusion\left({\bar{W}}_{\vec{w}\cdot \vec{x}},{\bar{W}}_{b}\right), \end{array}$$

where the fusion operator takes addition's place in the *net* calculation. The opinion of product of $\vec{w}$ and $\vec{x}$, ${\bar{W}}_{\vec{w}\cdot \vec{x}}$, is:

(4) $$\begin{array}{l} {\bar{W}}_{\vec{w}\cdot \vec{x}}={\bar{W}}_{\vec{w}}\cdot {\bar{W}}_{\vec{x}}. \end{array}$$

${\bar{W}}_{N}^{X}$ is calculated regardless of the activation functions since it doesn't make sense to apply activation functions on opinions. In this sense, our framework is not limited by the linearity or non-linearity of the neural network. During the training process, the weight and bias are updated during the backward propagation based on loss. Therefore, as shown in Figure 2, opinions of weight and bias should be updated simultaneously during the training as well. At the beginning, ${\bar{W}}_{\vec{w}}$ and ${\bar{W}}_{b}$ are initialized to have maximum uncertainty due to lack of evidence, and later on updated according to the neuron's output based on the same rule introduced in Equation (5).

Backward opinion of neuron ${\bar{W}}_{N}^{Y}$ in Equation (2) is an opinion from *Y* point of view hence it is calculated based on opinion of true label ${\bar{W}}_{y}$. In backpropagation, error is the key factor. Similarly, we use the error |*y*′ − *y*| in computation of ${\bar{W}}_{N|y}^{Y}$, the conditioned backward opinion of neuron, which is equivalent to ${\bar{W}}_{y^{\prime }|y}$, the opinion of neuron's output *y*′ given the true label *y*. During the training process, based on the opinion quantification from evidence rules introduced in section 3.2, if output of the neuron, *y*′, is in some tolerance region of true label *y*, i.e., there exists a small ϵ, s.t. |*y*′ − *y*| < ϵ, we count this as a positive evidence *r* to formulate the opinion of *y*′ given *y*: ${\bar{W}}_{y^{\prime }|y}$. Otherwise, it is a negative evidence *s*. Since the positive and negative evidences are calculated along with the training process, it will not cause extra computation expense. Intuitively, *r* and *s* represent the numbers of outputs that NN predicts correctly and wrongly, respectively, and they are updated during the training. After each update, ${\bar{W}}_{y^{\prime }|y}=\left(b_{y^{\prime }|y},d_{y^{\prime }|y},u_{y^{\prime }|y},a_{y^{\prime }|y}\right)$ is then formulated from these evidences according to the opinion quantification from evidence rules. After deriving the conditioned opinion, the backward marginal opinion of *N*, i.e., ${\bar{W}}_{N}^{Y}$, is calculated as follows (similar to the calculation of a marginal probability given a conditional probability):

(5) $$\begin{array}{l} {\bar{W}}_{N}^{Y}={\bar{W}}_{y^{\prime }}={\bar{W}}_{y^{\prime }|y}\cdot {\bar{W}}_{y}. \end{array}$$

### 4.4. Opinion Propagation in a General Topology

For a neural network (denoted as *NN*) with multiple inputs, multiple outputs, and multiple hidden layers, final opinion ${\bar{W}}_{NN}$ consists of opinions of all neurons in the final layer, i.e., all ${\bar{W}}_{neuron}$'s in output layer, each contains forward part and backward part, similarly to those of one neuron case. As shown in Figure 1B, for a neuron in a hidden layer, ${\bar{W}}_{neuron}^{H}$ is calculated as $fusion\left({\bar{W}}_{b},{\bar{W}}_{\omega_{1}}\cdot {\bar{W}}_{x_{1}},...\right)$. If it is the first hidden layer, then ${\bar{W}}_{input}={\left[{\bar{W}}_{x_{1}},{\bar{W}}_{x_{2}},...\right]}^{T}$ represents the opinions of data input. If it is the second or latter hidden layer, ${\bar{W}}_{input}$ represents the output opinions ${\bar{W}}_{neuron}$ from the previous layer. For a neuron in the output layer, the opinion is calculated as in Equation (2). The forward opinion ${\bar{W}}_{neuron}^{X}$ takes opinions of input (i.e., the output of previous hidden layer), opinions of weight, and opinions of bias into account. Similarly, ${\bar{W}}_{neuron}^{Y}$'s are the backward opinions of neurons in output layer, each of which is a function of opinions of true labels and opinions of neuron's output. After evaluating the opinions of all output neurons, ${\bar{W}}_{NN}$ is then calculated as the averaging opinion of all ${\bar{W}}_{neuron}$'s in output layer.

**Theorem 4.1**. Considering all neurons' opinions instead of only the neurons in the output layer causes opinion duplication.

Proof.Let us consider a simple neural network with one hidden neuron and one output neuron. According to the calculation strategy, the opinion of output neuron is calculated as:

$$\begin{array}{l} {\bar{W}}_{neuron}=fusion\left({\bar{W}}_{neuron}^{X},{\bar{W}}_{neuron}^{Y}\right)\\ \;=fusion\left(fusion\left({\bar{W}}_{b},{\bar{W}}_{\omega }\cdot {\bar{W}}_{x}\right),{\bar{W}}_{neuron}^{Y}\right), \end{array}$$

where ${\bar{W}}_{x}$ is the output opinion of the previous hidden neuron, i.e., ${\bar{W}}_{x}={\bar{W}}_{neuron}^{H}$. Therefore, the final opinion formula of the neuron in output layer reads:

$$\begin{array}{l} {\bar{W}}_{neuron}=fusion\left(fusion\left({\bar{W}}_{b},{\bar{W}}_{\omega }\cdot {\bar{W}}_{neuron}^{H}\right),{\bar{W}}_{neuron}^{Y}\right). \end{array}$$

If we take opinion of the hidden neuron ${\bar{W}}_{neuron}^{H}$ again into consideration when calculating the final opinion of this simple neural network, the final opinion equation of the neural network becomes:

$$\begin{array}{l} {\bar{W}}_{NeuronNetwork}=fusion\left({\bar{W}}_{neuron},{\bar{W}}_{neuron}^{H}\right)\\ \;=fusion\left(f\left({\bar{W}}_{neuron}^{H}\right),{\bar{W}}_{neuron}^{H}\right), \end{array}$$

where $f\left({\bar{W}}_{neuron}^{H}\right)$ represents that ${\bar{W}}_{neuron}$ is a function of ${\bar{W}}_{neuron}^{H}$. Hence, we can see the above equation counts the opinion of the hidden neuron twice and causes an opinion duplication. Since all the previous layers opinions are propagated to the final output layer, the opinion of the output neuron already takes the opinions of hidden neuron and input neurons into account. Which means we do not need to double count them again in the calculation of final opinion of the neural network.

**Figure 3:**
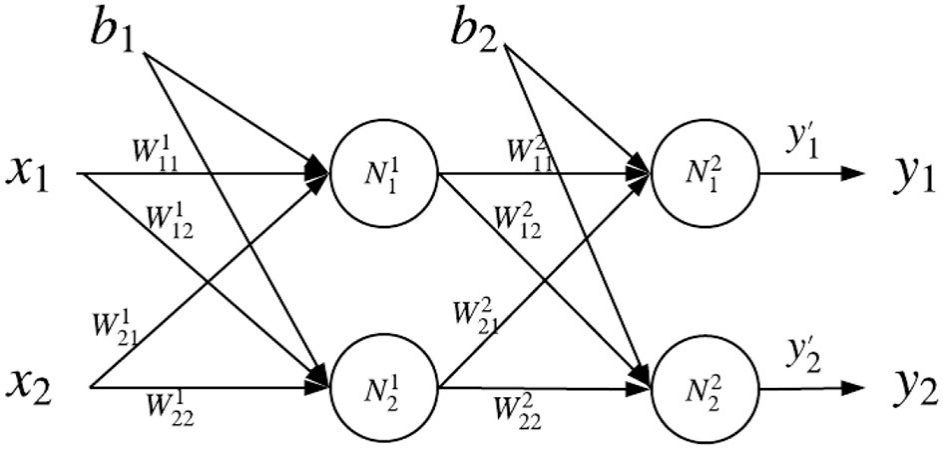
General topology example. The first hidden layer contains hidden neurons $N_{1}^{1}$ and $N_{2}^{1}$, and the second hidden layer contains hidden neurons $N_{1}^{2}$ and $N_{2}^{2}$.

Here we describe a concrete example of multi-layer neural network opinion evaluation by using DeepTrust. Let us derive an opinion of a neural network with two inputs, two hidden neurons, and two outputs, as shown in Figure 3. The final opinion of the neural network, ${\bar{W}}_{NN}$, is the fused opinion of all output neurons ($N_{1}^{2}$ and $N_{2}^{2}$):

(6) $$\begin{array}{l} {\bar{W}}_{NN}=fusion\left({\bar{W}}_{N_{1}^{2}},{\bar{W}}_{N_{2}^{2}}\right). \end{array}$$

${\bar{W}}_{N_{j}^{i}}$ is the opinion of *j*^*th*^ neuron in *i*^*th*^ layer. Opinion ${\bar{W}}_{N_{j}^{i}}$ is calculated by Equation (2): ${\bar{W}}_{N_{j}^{i}}=fusion\left({\bar{W}}_{N_{j}^{i}}^{X},{\bar{W}}_{N_{j}^{i}}^{Y}\right)$, more specifically:

(7) $$\{\begin{array}{l} {\bar{W}}_{N_{1}^{2}}=fusion({\bar{W}}_{N_{1}^{2}}^{X},{\bar{W}}_{N_{1}^{2}}^{Y}),\\ {\bar{W}}_{N_{2}^{2}}=fusion({\bar{W}}_{N_{2}^{2}}^{X},{\bar{W}}_{N_{2}^{2}}^{Y}), \end{array}$$

where the opinion of each neuron in output layer takes two parts into consideration: forward part from *X* point of view, and backward part form *Y* point of view. Since the forward part comes from the previous layers, the calculation formula of ${\bar{W}}_{N_{j}^{i}}^{X}$ is similar to Equation (3), with the multi-source fusion operator (Wang and Zhang, 2017):

(8) $$\{\begin{array}{l} {\bar{W}}_{N_{1}^{2}}^{X}=fusion({\bar{W}}_{b_{2}},{\bar{W}}_{w_{11}^{2}N_{1}^{1}},{\bar{W}}_{w_{21}^{2}N_{2}^{1}}),\\ {\bar{W}}_{N_{2}^{2}}^{X}=fusion({\bar{W}}_{b_{2}},{\bar{W}}_{w_{12}^{2}N_{1}^{1}},{\bar{W}}_{w_{22}^{2}N_{2}^{1}}),\\ {\bar{W}}_{N_{1}^{1}}^{X}=fusion({\bar{W}}_{b_{1}},{\bar{W}}_{w_{11}^{1}x_{1}},{\bar{W}}_{w_{21}^{1}x_{2}}),\\ {\bar{W}}_{N_{2}^{1}}^{X}=fusion({\bar{W}}_{b_{1}},{\bar{W}}_{w_{12}^{1}x_{1}},{\bar{W}}_{w_{22}^{1}x_{2}}). \end{array}$$

We can clearly see that ${\bar{W}}_{N_{1}^{2}}^{X}$ combines trust information of bias *b*_2_, weights $w_{11}^{2}$ and $w_{21}^{2}$, and neurons $N_{1}^{1}$ and $N_{2}^{1}$ in the previous layer. Backward opinion of each neuron in *NN*'s output layer in Equation (7) is calculated similarly to what Equation (5) states:

(9) $$\{\begin{array}{l} {\bar{W}}_{N_{1}^{2}}^{Y}={\bar{W}}_{N_{1}^{2}|y_{1}}\cdot {\bar{W}}_{y_{1}}\\ {\bar{W}}_{N_{2}^{2}}^{Y}={\bar{W}}_{N_{2}^{2}|y_{2}}\cdot {\bar{W}}_{y_{1}}. \end{array}$$

### 4.5. Opinion Quantification of NN's Output

DeepTrust not only has the ability to quantify the opinion of a NN in training phase under the assumption that the training data and the training process are accessible, but it can also be deployed in trust quantification of NN's decision or output when given a pre-trained neural network. This enables DeepTrust with wider usefulness and deeper impact in real world implementations. For most of the real-world situations, we only have access to a pre-trained NN, such as online machine learning models provided by cloud service providers, and we need to evaluate the trust of a given pre-trained NN and/or its output. To this end, in addition to the training phase, DeepTrust includes opinion and trustworthiness quantification in validation and test phases, under the assumption that the NN's topology is known. In validation phase, given a pre-trained NN (along with its topology) and validation data, DeepTrust can quantify opinion and trustworthiness of the pre-trained NN similarly as in section 4.4 in training phase. After calculating the opinion of the NN, the opinion of its output can be calculated similarly as the forward opinion of the NN, which is similar to generating a prediction in ordinary NN testing phase. Opinion and trustworthiness quantification of NN's prediction provides an evaluation of input data and NN inner working's trustworthiness, and is often useful when NN generates overconfident predictions. Beside accuracy, confidence value, this third dimension not only covers the objective data itself, but also consists NN and data's subjective trust information. Together with accuracy, a multi-objective optimization can be formed in future work.

## 5. Results and Findings

This section delivers two major experimental findings along with several minor remarks, and an application to show the usefulness of DeepTrust. Section 5.1 shows that different NN topologies have different opinions and trustworthiness, and DeepTrust has the ability to identify good topologies in terms of trustworthiness. We design a case study I that contains three parts and experiments on more than 25 NN topologies. In case study I we also find that accuracy and trustworthiness are not necessarily correlated. In section 5.2 we present that uncertainty *u* in opinion is not always malicious when it comes to trust quantification, whereas disbelief *d* hurts the trust the most. We design a case study II to experiment on six different cases to explore the impact of opinion data. In addition, we apply DeepTrust to 2016 presidential election predictors and show the usefulness of our work in section 5.3. We validate the network on MNIST and election dataset as they are well-studied and will help better understand the implications of DeepTrust. Finally, we apply DeepTrust to structures that form the building blocks of deep belief nets proposed by Hinton (Nair and Hinton, 2010) and show the batch-training trustworthiness evaluation process. Besides the architectures we experimented in this section, experiments indicate that DeepTrust is applicable to any other more complicated datasets and deeper neural network architectures.

### 5.1. A Good NN Topology Leads to High Projected Trust Probabilities, Even When Trained With Untrustworthy Data

Opinion and trustworthiness quantification of a multi-layered NN depends on the opinions about the dataset content, the training loss, and the network topology. Since the training loss is highly correlated with the network topology, in this work, we mainly focus on the effect of the opinion about the datasets content and the network topology. Given that the topology is more under the control of the designer, we first start with the impact analysis of the network topology.

#### 5.1.1. Case Study I: Experiment Setup

To investigate the relation among network topologies (i.e., we only vary the number of hidden layers and number of hidden units in each layer, other hyper parameters, such as the learning rate, activation functions are the same in all experiments), uncertainty degree of data, and opinion of a NN, we conduct a case study with three parts:

- Evaluate opinion of *NN*_1_ with topology 784-1000-10 and *NN*_2_ with topology 784-500-500-10, under original MNIST data with max belief opinion and damaged MNIST data with max uncertainty opinion assigned to the damaged data points. Data damage percentage ranges from 10 to 100%.
- Evaluate opinion of network with topology 784-x-10, where the number of hidden neurons x ranges from 100 to 2, 000, under original MNIST data and damaged MNIST data with 10–20% data damage. We evaluate in total 20 different topologies in this step.
- Evaluate opinion of NN with topology 784-{1000}-10, which represents the neural network with 784 neurons in input layer, 10 neurons in output layer, 1, 000 hidden neurons in total and distributed in 1 to 5 layers (more specifically, the topologies used are 784-1000-10, 784-500-500-10, 784-300-400-300-10, 784-250-250-250-250-10, and 784-200-200-200-200-200-10). The NNs are trained under original MNIST data and damaged MNIST data with 10–20% data damage.

The first part of case study I addresses the relation between opinion about a NN and uncertainty degree of data by comparing the opinion of different topologies under different data damage degree. A network topology regarded as among the best for the MNIST database (denoted by *NN*_1_), has a topology of 784-1000-10 (Simard et al., 2003) (where 784 neurons in the first layer are used for the input handwritten digit images, each with 28 ×28 pixels, the 1, 000 neurons in the second layer are used as hidden neurons, and the 10 neurons in the third layer are used for the 10 output classes, digit 0–9). To contrast the opinion quantification for *NN*_1_, we also consider *NN*_2_ with a topology of 784-500-500-10 (for which the neurons of the middle layer of *NN*_1_ are distributed equally to two hidden layers each of which with 500 neurons). To evaluate the opinion of *NN*_1_ and *NN*_2_, we first train them by feeding the training dataset once, one data point at a time, and then evaluate the opinion of trained networks based on their topologies and the training loss. To better realize the impact of topology, the NNs are trained with datasets with full confidence on the trustworthiness of data, i.e., the opinion about the dataset has the maximum belief to reflect minimum uncertainty of 0 regarding the dataset. We denote this maximum belief and zero uncertainty by {1, 0, 0, 0.5}, which represents *belief* = 1, *disbelief* = 0, *uncertainty* = 0, and *base rate* = 0.5. This helps our analysis to concentrate on the impact of topology only. In addition to evaluating the opinion of *NN*_1_ and *NN*_2_ with the highly trustworthy dataset, we also randomly take a subset of training data and flaw the labels by randomly altering them, and then feed the damaged training dataset to $NN_{1}^{D}$ and $NN_{2}^{D}$, which have the same corresponding topologies as those of *NN*_1_ and *NN*_2_, but with different parameters values (i.e., bias and weights) as the training data are different. Since the training set is damaged by altering some labels, the opinion of training set should be redefined accordingly to account for the damaged data. The opinion of damaged data point is set to be maximum uncertainty. More precisely, the opinion {0, 0, 1, 0.5} represents *belief* = 0, *disbelief* = 0, *uncertainty* = 1, and *base rate* = 0.5. The level of data damage is varied from 0 to 100%.

The second and third part of this case study investigate the relation between projected trust probability of NN and its topology. By varying the number of hidden neurons in one hidden layer and the number of hidden layers under same amount of hidden neurons as described above, we further explore the impact of topology on opinion and trustworthiness. Opinion setup for damaged data follows the same principle as that stated in the first part of this case study.

#### 5.1.2. Case Study I: Experimental Results

**Remark 5.1**. Higher percentage of damage in the input dataset results in higher levels of trust degradation, however the exact level is highly topology dependent. A good network topology leads to high trustworthiness even when trained with untrustworthy data.

**Figure 4:**
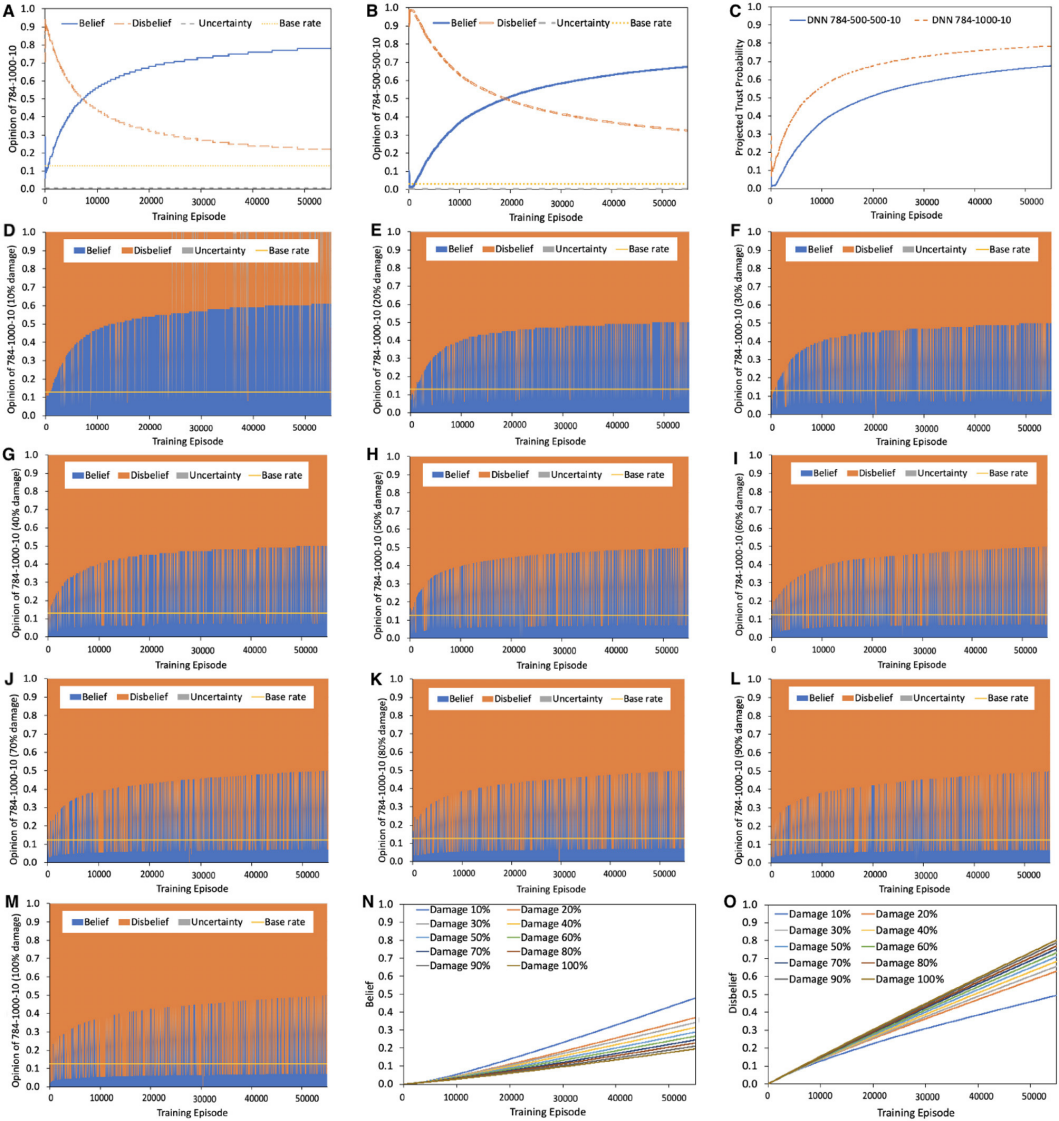
Opinion comparison between *NN*_1_ and *NN*_2_ under undamaged MNIST data and opinion of $NN_{1}^{D}$ under damaged MNIST data. **(A)** Opinion of *NN*_1_ with topology 784-1000-10. Belief reaches 0.78 when training data's uncertainty and disbelief are zero, i.e., belief is maximized. **(B)** Opinion of *NN*_2_ with topology 784-500-500-10. Belief reaches 0.68 when training data's belief is maximized. Base rate is calculated using computational strategy presented in section 4. **(C)** Projected trust probability comparison between *NN*_1_ and *NN*_2_. Topology impacts projected trust probability. More precisely, *NN*_1_ outperforms *NN*_2_ while both *NN*_1_ and *NN*_2_ are trained with same dataset (same opinion assigned to dataset: {1, 0, 0, 0.5}, and same training process). **(D–M)** $NN_{1}^{D}$ with the same topology as *NN*_1_, i.e., 784-1000-10, is trained with damaged data. Randomly take 10–100% training data, alter labels to introduce uncertainty and noise into dataset. Set opinion of damaged data point to have maximum uncertainty: {0, 0, 1, 0.5}. Belief is sparser while disbelief becomes denser in **(D–M)**, but there is still belief even the dataset is 100% damaged. **(N,O)** normalized cumulative belief and disbelief of $NN_{1}^{D}$ under 10% to largest data damage, averaged over 10 runs. Note that the cumulative belief is not zero and increases during training even for a completely damaged data. Also note that both disbelief and belief increase as a function of number of episodes.

Our experiments confirm this observation as shown in Figure 4. Figures 4A–C summarize the opinion and projected trust probability comparison between *NN*_1_ and *NN*_2_. The trust probability of *NN*_1_ and *NN*_2_ converge to 0.78 and 0.68, respectively. Figures 4D–M shows the opinion of $NN_{1}^{D}$. Training $NN_{1}^{D}$ with 10% data damage results in a belief value of 0.6. When damaged data percentage varies from 20 to 100%, the belief of *NN*_1_ converges to 0.5, with the disbelief increasing its portion as shown in Figures 4E–M. The opinion results of $NN_{2}^{D}$ are summarized in Supplementary Material Section 1, Figure S1. Training $NN_{2}^{D}$ with 10–100% data damage results in relatively lower belief value compared to $NN_{1}^{D}$. When damaged data percentage varies from 30 to 100%, the belief of $NN_{2}^{D}$ converges to 0.25. This confirms that for a robust topology on MNIST dataset, such as *NN*_1_, the impact of damage in the dataset is less severe as that in a NN with a frail topology (e.g., *NN*_2_) in terms of the belief and projected trust probability.

**Remark 5.2**. When choosing between two NN architectures, if the accuracy comparison doesn't provide good results, adding trustworthiness comparison into the performance measures helps with the decision making.

Accuracy comparison of *NN*_1_ ($NN_{1}^{D}$) and *NN*_2_ ($NN_{2}^{D}$) appears in Table 1. *NN*_2_ and most cases of $NN_{2}^{D}$ slightly outperform their corresponding *NN*_1_ ($NN_{1}^{D}$) cases in terms of accuracy. However, we believe this slight difference is not very convincing when choosing between these two different topologies. On the other hand, trust comparison acts as a more reliable tool, e.g., the impacted projected trust probability is almost 50% (49.99 vs. 25.1% for $NN_{1}^{D}$ and $NN_{2}^{D}$, respectively). We therefore propose quantifying opinions for NNs and using the opinion comparison among various NNs along with accuracy evaluation as a tool to determine the robustness of the NN topologies in both cases of trustworthy and untrustworthy data. Note that both topologies were trained by feeding all training data once, with one data point at a time. To increase the accuracy, a better training strategy is to feed the entire dataset to the NN multiple times, with a mini-batch of data at a time. DeepTrust works with batch-training as well.

**Remark 5.3**. Accuracy and trustworthiness are not necessarily correlated.

**Table 1:** Comparison of accuracy and projected trust probability between *NN*_1_ ($NN_{1}^{D}$) and *NN*_2_ ($NN_{2}^{D}$).

**Data damage percentage (%)**	**Accuracy (%)**		**Projected trust probability (%)**	
	**784-1000-10**	**784-500-500-10**	**784-1000-10**	**784-500-500-10**
0	90.29	91.03	78.46	67.54
10	81.18	80.74	60.74	44.71
20	71.77	73.53	50.00	30.34
30	67.90	65.22	50.00	25.09
40	61.31	60.51	50.00	25.09
50	52.60	53.15	49.99	25.10
60	50.46	52.15	49.99	25.09
70	46.33	44.40	49.99	25.09
80	40.08	40.40	49.99	25.09
90	35.02	38.94	49.99	25.09
100	36.10	37.19	49.99	25.10

**Figure 5:**
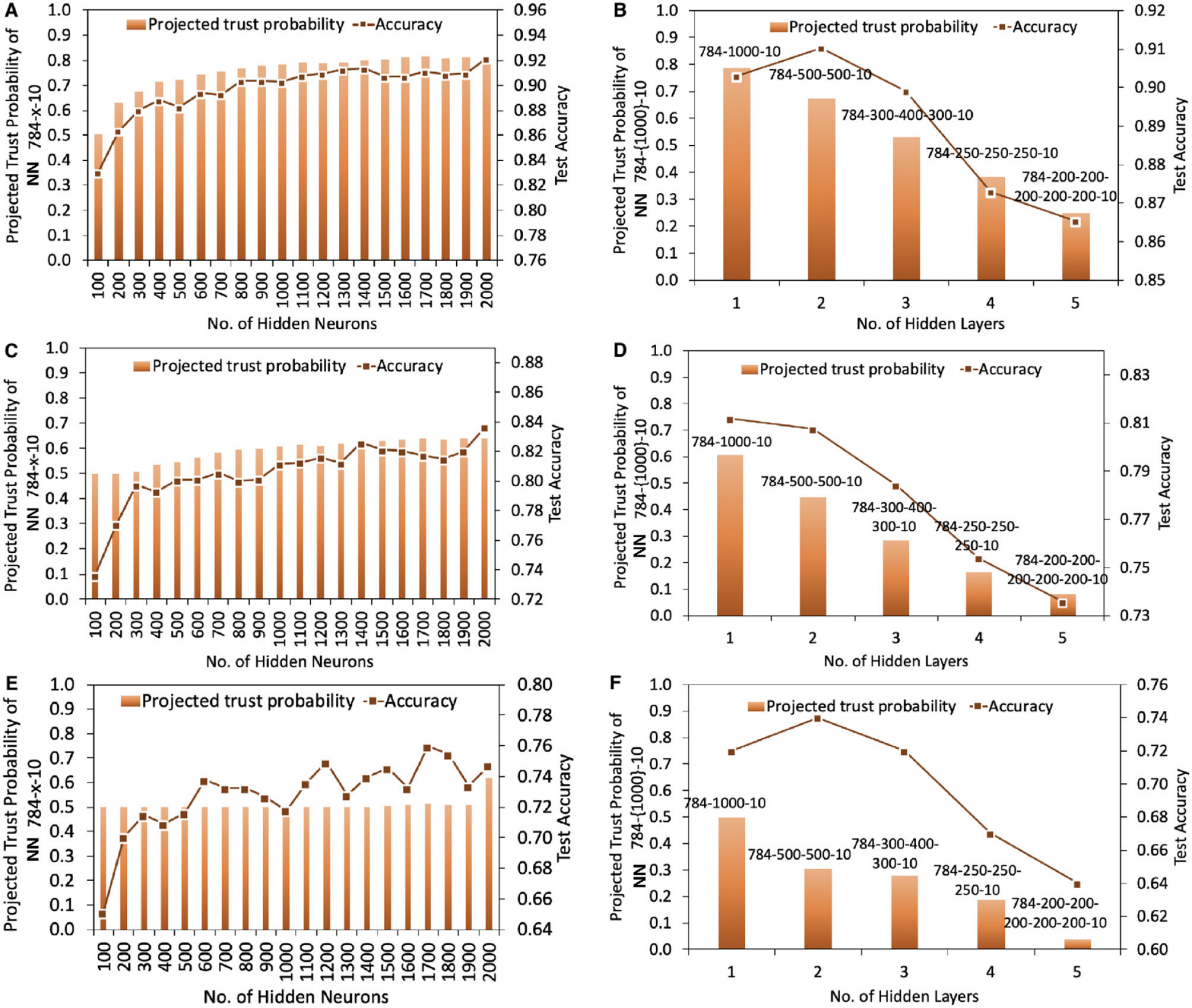
Projected trust probability and accuracy comparison of 784-x-10 and 784-{1000}-10 under original and damaged MNIST data. Training process in **(A,B)**, **(C,D)**, and **(E,F)** are under original MNIST data (with maximum belief and therefore zero uncertainty), also 10% and 20% data damage, respectively. **(A)** Projected trust probability of 784-x-10 reaches 0.8 when increasing the number of hidden neurons from 100 to 2, 000. Topology highly impacts the projected trust probability, especially when rearranging a certain number of hidden neurons, in various number of hidden layers. Accuracy hits the highest value with topology 784-2000-10, and the second best accuracy is given by 784-1400-10. **(B)** Compared to other topologies, projected trust probability of 784-1000-10 is the highest with value 0.78, while topology 784-500-500-10 outperforms others in terms of accuracy. **(C)** Under 10% data damage, projected trust probability of 784-x-10 reaches 0.64 when increasing the number of hidden neurons from 100 to 2, 000. **(D)** Topology 784-1000-10 outperforms others in both accuracy and trust. **(E)** Under 20% data damage, projected trust probability of 784-x-10 settles at 0.5 when increasing the number of hidden neurons from 100 to 1, 900, while 784-2000-10 provides highest trust probability. **(F)** Topology 784-1000-10 results in highest trust probability, while topology 784-500-500-10 reaches highest accuracy.

Figure 5 shows the projected trust probability and accuracy comparison of 784-x-10 and 784-{1000}-10 trained under original MNIST data with maximum belief opinion {1, 0, 0, 0.5}, and under 10 and 20% data damage (maximum uncertainty opinion, i.e., {0, 0, 1, 0.5}). We take low data damage percentage here because in real life severely damaged data will not be used to train the models at all. The results confirm that data damage impacts both trustworthiness and accuracy, however accuracy and trustworthiness are not necessarily correlated, i.e., topologies that result in highest accuracy, may not reach the highest trustworthiness levels. Under original and slightly damaged MNIST data, adding more hidden neurons results in higher projected trust probabilities when the number of layers is fixed, however the trust probability increasing rate tends to slow down as more neurons are added. When the data damage is higher, e.g., 20%, adding more neurons in one layer doesn't lead to a significant increase in the trust outcome. On the contrary, while keeping the total number of hidden neurons as fixed, changing the number of hidden layers strongly impacts the projected trust probability. Therefore, when the dataset is damaged and the training resource is limited, varying the number of hidden layers rather than number of hidden neurons is a more efficient strategy to obtain higher levels of projected trust probability outcome.

### 5.2. Uncertainty Is Not Always Malicious When Evaluating the Opinion and Trustworthiness of A Neural Network

In SL, the lack of confidence in probabilities is expressed as uncertainty mass. Uncertainty mass represents lack of evidence to support any specific value (Jøsang, 2016). An important aspect of uncertainty quantification in the scientific literature is statistical uncertainty. This is to express that the outcome is not known for each time we run the same experiment, but we only know the long-term relative frequency of outcomes. Note that statistical uncertainty represents first-order uncertainty, and therefore is not the same type of uncertainty as the uncertainty mass in opinions, which represents second-order uncertainty (Jøsang, 2016). The impact of the topology to opinion quantification of a NN is discussed in the previous section, and the impact of opinion of data is explored as follows.

#### 5.2.1. Case Study II: Experiment Setup

To address the impact of opinion of data to opinion and trustworthiness of a neural network, a case study is designed as follows:

- Construct a simple neutral network $NN_{1}^{S}$ with topology 3-1-1. Set opinion of training dataset to be six cases: max belief, max disbelief, max uncertainty, neutral, equal belief & disbelief, and more belief than disbelief. Detailed opinion setup is described in Case I to Case VI.
- Construct another simple neutral network $NN_{2}^{S}$ with same setup and same training data as $NN_{1}^{S}$, but $NN_{2}^{S}$ has 10 hidden neurons. $NN_{2}^{S}$ uses a more complicated topology, i.e., 3-10-1, to realize same function as $NN_{1}^{S}$.

Both neural networks are trained under same process and the opinions of both are evaluated after training. This case study focuses on the impact of opinion of data, hence simple topologies are chosen without loss of generality. Starting with zero bias may at times generate better outcomes than cases with neutral information. To present the true meaning of this statement, we evaluate the impact of the degree of opinion confidence in the training dataset on the opinion and trustworthiness of NN. We consider the following six cases of opinion for the training dataset:

- Case I—Max belief: set the opinion of training dataset *Opinion*_*Data*_ to be maximum belief, i.e., {1, 0, 0, *a*}. This means the training of NN is performed with the highest level of data trustworthiness.
- Case II—Max disbelief: *Opinion*_*Data*_ is set to be maximum disbelief, i.e., {0, 1, 0, *a*}, which means that the dataset is untrustworthy.
- Case III—Max uncertainty: *Opinion*_*Data*_ is set to be maximum uncertainty, i.e., {0, 0, 1, *a*}. This setting is used when we do not know whether we can trust the dataset due to lack of information.
- Case IV—Neutral: *Opinion*_*Data*_ is set to be neutral: {1/3, 1/3, 1/3, *a*}. This is similar to Case III in the sense that we lack information on the dataset, however it presents scenarios where the levels of uncertainty and trustworthiness of data are in the same level.
- Case V—Equal belief & disbelief: *Opinion*_*Data*_ is set to be {0.5, 0.5, 0, *a*}. This opinion represents that the belief mass and disbelief mass are both equal to 0.5 with minimum uncertainty of 0, which is the scenario that an agent cannot generate a certain opinion, but there is no uncertainty formulation.
- Case VI—More belief than disbelief: *Opinion*_*Data*_ is set to be {0.75, 0.25, 0, *a*} to compare with Case III and further investigate the importance of uncertainty in opinion quantification. This setting contains three times more belief mass than disbelief and zero uncertainty.

All base rates are set to be 0.5 to represent an unbiased background. Note that cases III and IV are more realistic, whereas cases I and II are more on the extreme sides.

#### 5.2.2. Case Study II: Experimental Results

**Remark 5.4**. Disbelief hurts the trust the most while uncertainty is not always malicious.

**Figure 6:**
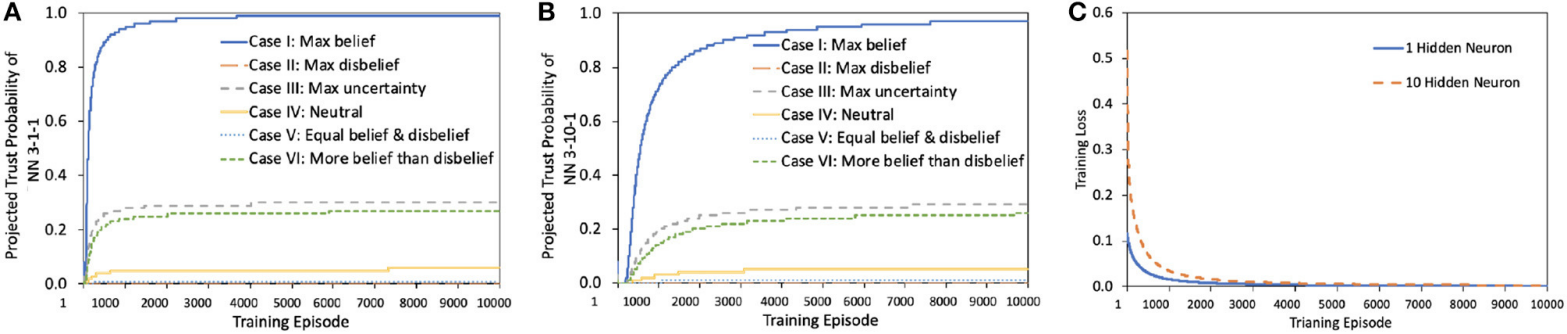
Projected trust probability and loss comparison of $NN_{1}^{S}$ and $NN_{2}^{S}$. **(A,B)** Projected trust probability comparison of $NN_{1}^{S}$ and $NN_{2}^{S}$ in afore-mentioned cases. Both $NN_{1}^{S}$ and $NN_{2}^{S}$ are trained under same process with same dataset. Training loss comparison is shown in **(C)**. The results of $NN_{1}^{S}$ and $NN_{2}^{S}$ are similar. $NN_{1}^{S}$ and $NN_{2}^{S}$ reach a certain trust probability level with different speeds, more precisely, $NN_{1}^{S}$ reaches a desired projected trust probability level faster.

Our experimental results in Figure 6 confirm that for the same topology and training loss, the afore-mentioned cases generate different levels of projected trust probability in the outcome of the trained NN. According to our experiments, NN in Case III results in much higher projected trust probability than in Case IV, V, and VI, which leads to the conclusion that disbelief of training dataset hurts the final trust probability of NN more than uncertainty. In addition, lack of uncertainty measurement in Case V and VI leads to low projected trust probability, however, this dilemma occurs frequently in real world. It is therefore recommended to set the opinion of dataset to maximum uncertainty in cases where belief, disbelief, and uncertainty are at similar levels, due to lack of information. Furthermore, if an AI decision maker cannot provide a result with full confidence, uncertainty mass is recommended to be generated along with the decision.

We use two simple neural networks, $NN_{1}^{S}$ and $NN_{2}^{S}$, both of which implement a function that do binary OR on the first two inputs and ignore the third input, using the 3-1-1 and 3-10-1 topologies, respectively. The simplicity of the function helps us focus on the impact of the opinion of dataset, as both $NN_{1}^{S}$ and $NN_{2}^{S}$ would very well implement it, given sufficient number of training episodes. The projected trust probability comparison is illustrated in Figure 6. The projected trust probabilities of trained $NN_{1}^{S}$ and $NN_{2}^{S}$ are different in six cases as expected:

- Case I: Both $NN_{1}^{S}$ and $NN_{2}^{S}$ are highly trustworthy for such a scenario. The reasons are 2-fold: First of all, the simple topologies of $NN_{1}^{S}$ and $NN_{2}^{S}$ are sufficient to realize the simple two-input OR functionality. This is confirmed by the fact that belief sharply saturates to maximum and the training loss drops to minimum. Secondly, training is done with a trustworthy dataset, which contains no noise, glitch or uncertainty.
- Case II: Both $NN_{1}^{S}$ and $NN_{2}^{S}$ are untrustworthy because of the highly untrustworthy dataset. Therefore, the trust in the outcome of the network shows maximum disbelief establishment.

**Remark 5.5**. Use data with maximum uncertainty opinion to train a NN, then belief mass of this pre-trained NN can be non-zero.

- Case III: The results for this case are depicted in Figures 6A,B and in Supplementary Material Section 1, Figure S2. They confirm that after all, there is hope for belief, even in the case of maximum uncertainty. Detailed opinion results in terms of belief, disbelief, uncertainty, and base rate appear in Figure S2. Belief can be extracted from total uncertainty as illustrated in Figures S2C,I. The opinions of $NN_{1}^{S}$ and $NN_{2}^{S}$ have non-zero belief values, even when the opinion of dataset is set to have maximum uncertainty. This result is helpful in data pre-processing: uncertain data should not be filtered out since uncertainty has its own meaning. Even if the data is fully uncertain, there is still hope after all for belief.
- Case IV: The opinion of training dataset is neutral {1/3, 1/3, 1/3, 0.5}, which means belief, disbelief, and uncertainty of the dataset are set to be equal. This neutrality in terms of similar levels of belief, disbelief, and uncertainty in dataset can damage the projected trust probability in the outcome. The results shown in Figure 6 confirm lower levels of projected trust probability when compared to Case III, which leads to the conclusion that if no information about dataset is given, starting with total uncertainty is actually better than with biased opinion, and even a neutral one.
- Case V: In such scenarios, the dilemma of belief and disbelief is brought to maximum when *belief* = *disbelief* = 0.5 with zero uncertainty. The results in this case settle in much lower belief and projected trust probability values compared to those of Case III and IV. This reveals that the neutral case with uncertainty as in Case IV is much better than this neutral case without uncertainty measurement.
- Case VI: The results in this case are comparable to those in Case III in terms of trust since the belief mass of training data is three times more than disbelief, and the belief contributes the most to the results. However, lack of uncertainty measure in this uncertain case (there exists both belief and disbelief, and none of them plays the major role) leads to low projected trust probability in the end. Although similar to those of $NN_{1}^{S}$, the results of $NN_{2}^{S}$ show lower projected trust probability levels upon convergence for cases I, III, IV and VI, while showing the same level of convergence, but slower rate for the rest of the cases.

### 5.3. Did You Trust Those Who Predicted Trump to Lose in 2016 Election?

A significant amount of machine learning related projects or research activities involve utilization of pre-trained NNs. Training process is time consuming, expensive, or even inaccessible. In any case, one crucial question to answer is how much we should trust in the predictions offered by those pre-trained NNs. DeepTrust may shed light on this by providing opinion and trustworthiness quantification of NN's prediction. To further show the usefulness of DeepTrust, we apply DeepTrust on 2016 election prediction and quantify opinion (and projected trust probability) of the two major predictions back in 2016: “Trump wins” and “Clinton wins.”

**Figure 7:**
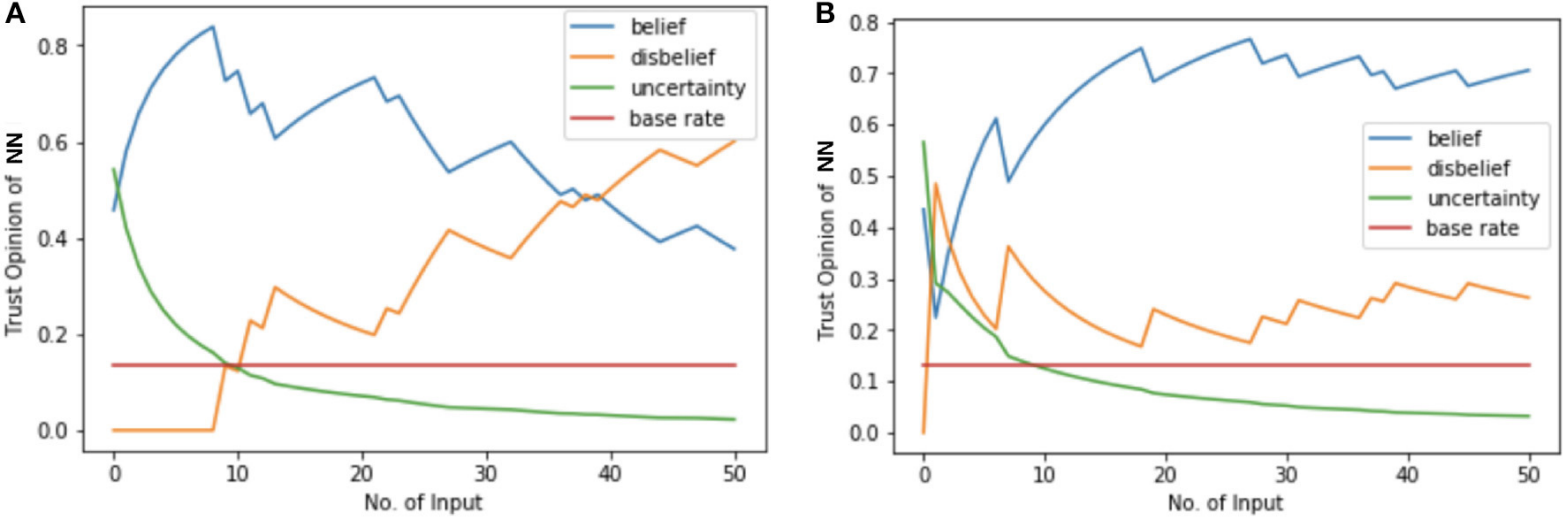
NN opinion results of 2016 election prediction. **(A,B)** Opinion comparison of presidential election predictors NN 1-32-32-1 and NN 9-32-64-32-1. **(A)** NN 1-32-32-1 is trained under original pre-election poll data. The projected trust probability of this NN in validation phase reaches 0.38, and its opinion reaches {0.38, 0.60, 0.02, 0.13}. **(B)** NN 9-32-64-32-1 is trained under enriched pre-election poll data. The opinion of this NN is {0.71, 0.26, 0.03, 0.13}, which has higher belief value and results in more trustworthy predictions.

2016 presidential election prediction has been called “the worst political prediction” and the erroneous predictions hand us an opportunity to rethink AI political prediction. In this case study we use 2016 presidential pre-election poll data (FiveThirtyEight, 2016), which contains more than 7000 state-wise pre-election polls conducted by CNN, ABC News, Fox News, etc. We train a NN with structure of 1-32-32-1 to predict the winner between Hillary Clinton and Donald Trump. Input of the NN is state and the output is Clinton vs. Trump. The training accuracy saturates to 63.96% and the trained NN predicts Clinton as president by winning 38 states and 426 votes, which is consistent with most of the presidential election predictors back in 2016. To quantify the opinion and trustworthiness of this trained NN, we use the 2016 presidential election results in the validation phase based on the assumptions that (i) we are given a trained NN which predicts Clinton to win the election, (ii) the election data has the maximum belief opinion of {1, 0, 0, 0.5} because the true election result is a trustworthy fact. The opinion of the NN 1-32-32-1 is shown in Figure 7A. In validation phase, the projected trust probability of this NN is 0.38 with low belief value of 0.38. After calculating the opinion in validation phase, opinion of this NN's output is quantified in test phase. Input of the NN in test phase has maximum belief value because of the maximum trustworthiness of voters in real election. The opinion of NN's output is {0.21, 0.77, 0.02, 0.03}, which results in 0.21 projected trust probability. By utilizing DeepTrust, the opinion and trustworthiness of this NN presidential election predictor is quantified and we show that its output, “Clinton wins presidential election,” is untrustworthy.

To further show the usefulness and effectiveness of DeepTrust, we quantify the opinion and trustworthiness of a NN which predicts Trump as winner in presidential election, and verify that this result is more trustworthy. The 1-32-32-1 NN predictor results in wrong prediction because of the untrustworthy pre-election poll data. Multiple factors related to pre-election poll data, such as “shy Trumpers,” lack of voters' detailed information, such as race, sex, education, income, etc. might have resulted in untrustworthiness and uncertainty in the whole prediction process. To present that this could have been avoided, had DeepTrust been used, we enrich the dataset by adding afore-mentioned detailed information of voters, and construct a dataset with 9 features: state, poll sample size, percentage of black, white, Latino, male, female, percentage of bachelor degree, and average household income. The NN predictor we use for this enriched dataset has the structure of 9-32-64-32-1, and its training accuracy reached 67.66% and predicts Trump as winner by wining 36 states and 336 votes. This result is more accurate than the previous one and closer to the true 2016 election result. This 9-32-64-32-1 NN should be more trustworthy, and we show that DeepTrust verifies this claim. The opinion of NN 9-32-64-32-1 is quantified in validation phase, and the results are shown in Figure 7B. The projected trust probability of this NN reaches 0.71 with 0.70 belief value. In testing phase, the opinion of this NN's output, “Trump wins presidential election,” is {0.50, 0.46, 0.04, 0.01} with 0.5 projected trust probability, which verifies that this result is relatively more trustworthy than previous “Clinton wins” result given by the 1-32-32-1 NN.

### 5.4. Trustworthiness Quantification With Batch Training

**Figure 8:**
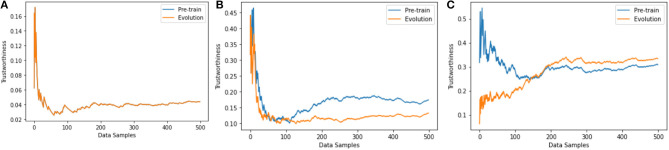
Trustworthiness comparison among three training strategies. **(A)** After one epoch of sample by sample training, the accuracy is 20.11%. The neural network is underfitting and stable, i.e., the loss is not decreasing in the first epoch of sample by sample training. The trustworthiness results from “pre-train” and “evolution” versions are the same. **(B)** After one epoch of mini-batch training, the accuracy is 47.23%. The neural network is underfitting and very unstable. Evaluating the trustworthiness on more data samples minimizes the gap between “pre-train” and “evolution” versions. **(C)** After multiple epochs of mini-batch training the accuracy achieves 59.49%. The neural network is semi-stable and the trustworthiness evaluation results between the two versions get closer if more data samples are used for trustworthiness evaluation.

In previous sections, we apply our framework to a number of neural network architectures and evaluate trustworthiness during the training process. We train networks and evaluate trustworthiness sample by sample to make clear observation of trustworthiness evolution during training. In this section, we quantify trustworthiness of a neural network training with mini-batches of data. We follow Hinton and Bengio (Nair and Hinton, 2010; Glorot et al., 2011) and use the neural network architecture proposed for the NORB dataset (LeCun et al., 2004). The neural network providing the best results on NORB has 4, 000 units in the first hidden layer and 2, 000 in the second. The activation function applied to all hidden units is ReLU. We follow Hinton's data pre-processing strategy to down sample the images to 32 ×32 and train with batch size 128. We compare the trustworthiness of the neural network under three training strategies: (i) one epoch of sample by sample training, (ii) one epoch of mini-batch training, (iii) multiple epochs of mini-batch training until stable. The results are shown in Figure 8. Compared to sample by sample training, batch training shows advantages in both accuracy and trustworthiness. The trustworthiness of the neural network in (i) and (ii) are clearly lower than in (iii) because the performance is not saturated with only one epoch of training. For the same neural network architecture, the trustworthiness and accuracy are positively correlated. In addition to evaluating trustworthiness along with the training process as in previous layers (to see how the trustworthiness evolves through the training process), we also pre-train the neural network, and then use pre-trained neural network to make predictions on all training samples. We use the prediction results to calculate the error terms |*y* − *y*′| and calculate the trustworthiness of the network. We refer these two trustworthiness quantification versions as “evolution” and “pre-train” and compare them in Figure 8 as well. These two versions get close to each other when evaluating on more data samples. These results prove the stability of our calculated trustworthiness and inform that if we have a pre-trained neural network, we can approximately quantify the trustworthiness by feeding in a large amount of data samples to the pre-trained neural network without the need of knowing the training process. This conclusion provides justification of our framework design in section 4.5.

## 6. Discussion

The increasing application of AI raises concerns regarding the security and morality of AI. Questions, such as “Why should I trust AI,” “How much should I trust AI,” “What are the chances my trust in AI, may result in tragic consequences” become daily concerns. Quantification of uncertainty and trust has become a popular research topic due to the growing applications of AI. However, the lack of theory and data becomes the major obstacle of trust quantification. In this work, we address the trust issues of neural networks by proposing DeepTrust, a framework to quantify opinion and trustworthiness of multi-layered NNs based on subjective logic, a formalism for representing and reasoning under probabilistic information.

A recent survey defines trustworthiness as certification and explanation (Huang et al., 2018). Certification techniques improve the human users' confidence on the correctness of the NNs, and the explanation techniques increase human users' understanding about the NNs and thus improve the trust (Huang et al., 2018). Research efforts either focus on certification or explanation. DeepTrust provides a novel angle of quantifying trust of the NNs by utilizing a formal trust metric, SL. DeepTrust is a combination of certification and explanation. The opinion quantification of NNs in training process is related to the correctness of NNs' output and the quality of the topology and training data. For pre-trained NNs, the opinion quantification is evidence-based, where the correct output votes for positive evidence and wrong output votes for negative evidence.

DeepTrust imports SL to AI and to the best of our knowledge, DeepTrust is the first work to quantify the opinion and trustworthiness of multi-layered NNs based on opinion about data and topology of the neural network. We find that the opinion and trustworthiness of NN is affected by both the topology and trustworthiness of the training data, and some topologies are more robust than others. More precisely, a robust topology results in higher projected trust probability values, not only when trained with trustworthy data, but also when fed with untrustworthy data. In extreme cases where only uncertain data is available, belief can still be extracted out of pure uncertainty. Designing NNs is generally a challenging task. We propose to adjust the topology, i.e., number of hidden layers, number of hidden neurons in hidden layers, etc., according to opinion and trustworthiness, and along with accuracy, subject to various costs, such as training time, and memory space limits. Whenever there is a trade-off between accuracy and trustworthiness, we recommend considering both in most cases and weight trustworthiness more in safety and security related applications. Based on our observations, accuracy and trustworthiness of the outcome do not necessarily correlate. DeepTrust may therefore shed light to the design of NNs with focus not only on accuracy but also the trust, while dealing with untrustworthy datasets. Further, DeepTrust can be used to quantify opinion and trustworthiness of pre-trained NNs and their output in various applications.

One limitation of our work is that DeepTrust quantifies trustworthiness of neural networks based on the opinions of data. However, the trust information about data is not always available mostly because the data collectors may not take the responsibility to provide such information. We plan to solve the trustworthiness of neural networks in two steps, namely model trustworthiness and data trustworthiness. In this work, we propose to solve the model trustworthiness by DeepTrust based on the availability assumption of data trustworthiness. Therefore, we would like to reveal the significance of the data trustworthiness and we believe that machine learning and AI researchers need to take one step forward when collecting the data. Not only the data value itself is important, the trustworthiness of the data is also a significant factor. However, the existing data collection process misses that. To solve this contradiction, as part of our follow-up work we will develop a quantifier for data trustworthiness to further enhance the whole trustworthiness quantification of neural networks. One way to relax the data trustworthiness availability assumption is to assume a maximum uncertainty of data, which provides a neutral evaluation as discussed in section 5.2.

In this work, we applied DeepTrust to quantify trustworthiness of neural network architectures with multiple hidden layers and non-linear activation functions. DeepTrust is applicable to deeper NNs and our future work will incorporate neural networks with convolutional layers and pooling layers such that we can test on more popular deep neural network architectures. DeepTrust applies to both classification and regression problems since the value of input and output does not affect the calculation of the opinions. As long as we have true labels, i.e., in the realm of supervised learning, DeepTrust can calculate the trustworthiness of the model and the output. The major assumption in SL is that collecting the evidence (positive or negative) reduces the uncertainty since the second-order uncertainty in SL represents the vacuity of evidence. A case we would like to discuss is that, intuitively, it looks like when we collect some evidences, the uncertainty mass should be decreasing. However, the evidence could be useless and should not contribute to either belief or disbelief mass. This is one of the shortages of monotonic logics, i.e., learning a new piece of information cannot reduce what is known. SL is not monotonic logic and it can handle belief revision. This case of “useless evidence” can be handled by the trust revision method in SL, which are designed to handle cases where the sources are unreliable. To apply the mapping from evidence to opinion, we assume that the sources are reliable, and every evidence contributes to either belief or disbelief. Another potential and intuitive way to solve this “useless evidence situation” is to modify SL by adding in a new type of evidence to contribute to uncertainty mass. However, this approach needs a strict and careful mathematical proof to make sure it is consistent with the existing syntax of SL, we will explore this path in the future as an additional contribution to SL.

## Data Availability Statement

Publicly available datasets were analyzed in this study. This data can be found here: https://www.kaggle.com/fivethirtyeight/2016-election-polls.

## Author Contributions

MC, PB, and SN contributed to the design of the research including simulations and experiments, contributed to the writing/revision of the manuscript. MC contributed to the implementation of simulator, running experiments, preparing the figures and tables, and their captions and organizing the information in the main text and also Supplementary Material. All authors contributed to the article and approved the submitted version.

## Conflict of Interest

The authors declare that the research was conducted in the absence of any commercial or financial relationships that could be construed as a potential conflict of interest.
