# Supplementary material for: There Is Hope After All: Quantifying Opinion and Trustworthiness in Neural Networks
Source: Front Artif Intell. 2020 Jul 31;3:54. doi: 10.3389/frai.2020.00054 (PMC7861320; doi:10.3389/frai.2020.00054)
Supplement: Supplementary file 1 [file Data_Sheet_1.PDF]

Supplementary Materials

1 ADDITIONAL EXPERIMENTAL RESULTS

Additional experimental results is presented in this Section. Fig. S1 shows opinion results of NN_2^D. Training NN_2^D with 10% and 20% data damage results in belief value compare to NN_1^D 0.4 and 0.3, respectively. When damaged data percentage varies from 30% to 100%, the belief of NN_2^D converges to 0.25, with the disbelief increasing its portion as shown in Fig. S1C-J. This confirms that for a robust topology such as NN_1, the impact of damage in the dataset is less severe as that in a NN with a frail topology (e.g., NN_2) in terms of the belief and projected trust probability. Fig. S2 shows the opinion comparison of trained NN_1^S and NN_2^S under six cases stated in Findings. The results of case III in Fig. S2C, I confirm that belief can be extracted from total uncertainty.

2 SUBJECTIVE TRUST NETWORK

The state of the world can be represented by various logic and probabilistic reasoning formalisms, such as binary logic and probability calculus to reflect the duality between the assumed objective reality and the perceived subjective world. The true or false state in binary logic fits well in an assumed objective reality, and probabilities ranging in $[0, 1]$ reflect subjectivity by allowing propositions to be partially true. However, estimating probabilities with confidence cannot be achieved and thus arguments such as "I don't know" cannot be expressed in either binary logic or probabilistic logic. Hence subjective logic (SL), was presented by A. Josang, which extends probabilistic logic by explicitly including degrees of uncertainty and vagueness (Jøsang, 2016).

To gain some intuition behind the proposed approach to quantify the opinion of NNs, let us consider an abstract yet simplified case study of a small subjective trust network as in Fig. S3. We aim to quantify the opinion when dealing with competitive opinions using SL concepts (e.g., arguments in SL are subjective opinions of a state). To evaluate analyst A's opinion of variable X, source B and source C's opinions are used as advice since analyst (user) A doesn't hold direct path to variable X. This relationship is common in social networks, where an analyst A may want to purchase an object X, or assess an object X (e.g., in a poll), but he/she doesn't have direct information about this object, so A has to rely on other buyers' and users' advice or reviews to develop an opinion and take action (e.g., purchase or vote for object X). Source B and C's opinion about variable X is represented as $\overline{W}_X^B$ and $\overline{W}_X^B$, respectively. In SL, an opinion contains belief b, disbelief d, uncertainty u, and base rate a. The projected trust probability is calculated as $b + u * a$. Since A has to count on these sources to collect information about X, A's opinion to B and C, $\overline{W}_B^A$ and $\overline{W}_C^A$, are used to derive A's opinion about X, i.e., $\overline{W}_X^A$, which is calculated as $fusion(\overline{W}_X^{[A;B]}, \overline{W}_X^{[A;C]})$. $\overline{W}_X^{[A;B]}$ and $\overline{W}_X^{[A;C]}$ represent A's opinion about X through source B and C, respectively, and $fusion(opinion_1, opinion_2, ...)$ represents the fusion operator to combine opinions. $\overline{W}_X^{[A;B]}$ is calculated by trust discounting, a multinomial calculation is presented (Jøsang, 2016):

$$\begin{cases} \vec{b}_X^{[A;B]}(x) = \vec{p}_B^A \vec{b}_X^B(x), \\ u_X^{[A;B]} = 1 - \vec{p}_B^A \sum \vec{b}_X^B(x), \\ \vec{a}_X^{[A;B]}(x) = \vec{a}_X^B(x). \end{cases} \tag{S1}$$

In the presence of multiple sources of opinions with various levels of belief and at times conflicting opinions, providing the means of averaging or cumulative fusion of those opinions has been shown to produce a better belief system than one with belief opinions in isolation (Jøsang, 2016; Wang et al., 2017).

REFERENCES

Jøsang, A. (2016). *Subjective logic* (Springer)

Wang, D., Zhang, J., et al. (2017). Multi-source fusion in subjective logic. In *Information Fusion (Fusion), 2017 20th International Conference on* (IEEE), 1–8

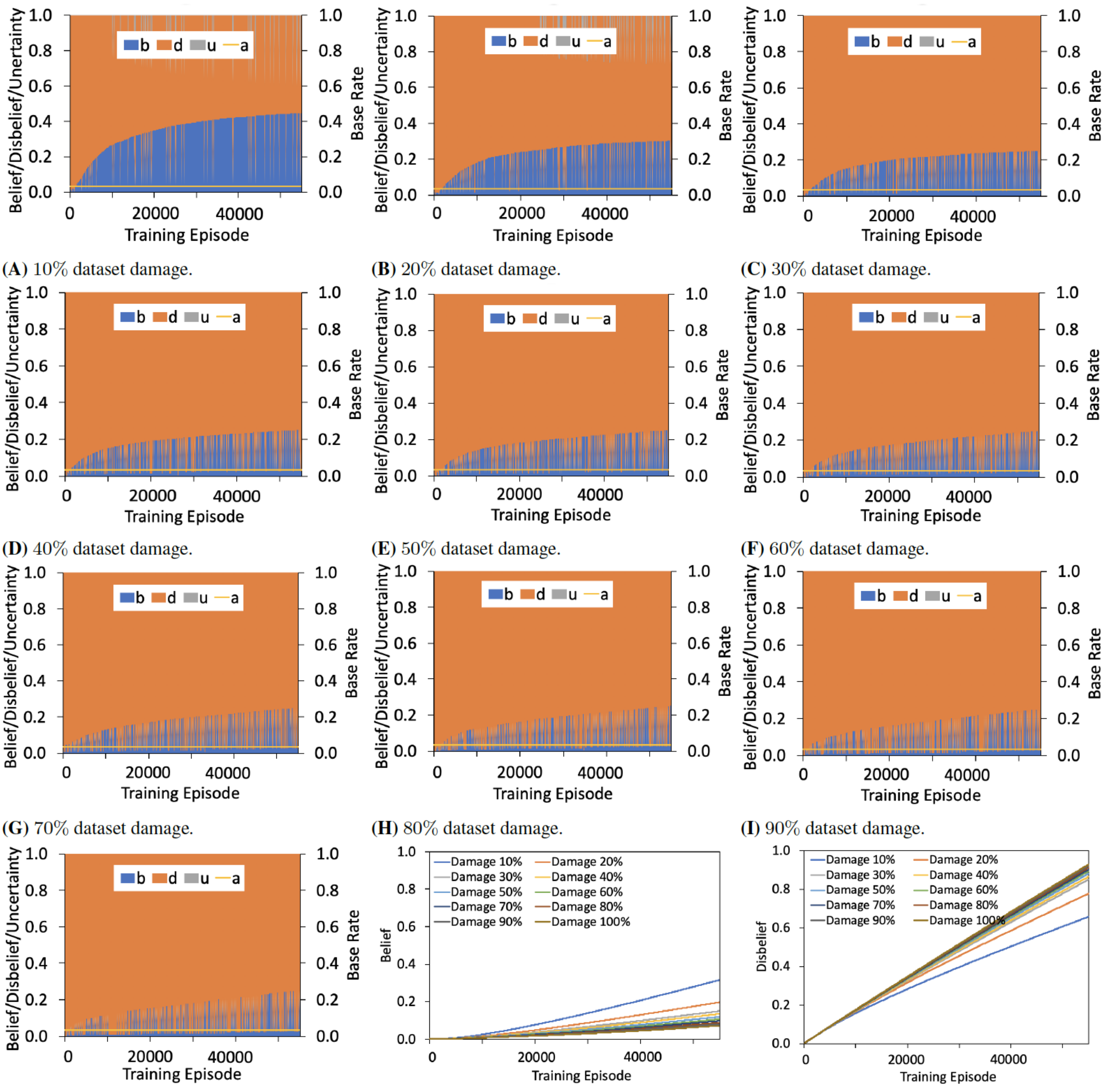

(A) 10% dataset damage. **(B)** 20% dataset damage. **(C)** 30% dataset damage. **(D)** 40% dataset damage. **(E)** 50% dataset damage. **(F)** 60% dataset damage. **(G)** 70% dataset damage. **(H)** 80% dataset damage. **(I)** 90% dataset damage. **(J)** 100% dataset damage. **(K)** Belief. **(L)** Disbelief.

Figure S1. Opinion comparison of NN_2^D for MNIST with 10% to 100% of data damage. A-J, NN_2^D with same topology as that of NN_2, i.e., 784-500-500-10, is trained with damaged data. Dataset is damaged by randomly taking a subset of training data, alter labels to introduce uncertainty and noise. Opinion of damaged data point is set to have maximum uncertainty: $(0, 0, 1, 0.5)$. b, d, u, a represent belief, disbelief, uncertainty, and base rate, respectively. As the level of data damage increases, the belief in outcome becomes sparser, but there is still belief even if the dataset is 100% damaged. **K-L,** normalized cumulative belief and disbelief of NN_2^D under 10% to 100% data damage. Compared with NN_1^D in the main text, belief settles at lower value when same portion of data is damaged, this is because the 784-500-500-10 topology is not as robust as the 784-1000-10 topology for MNIST database.

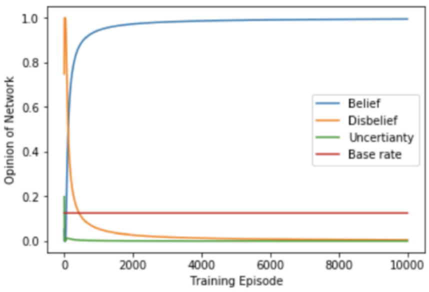

(A) Opinion of NN_1^S in Case I

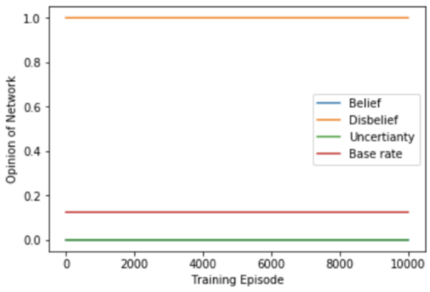

(B) Opinion of NN_1^S in Case II

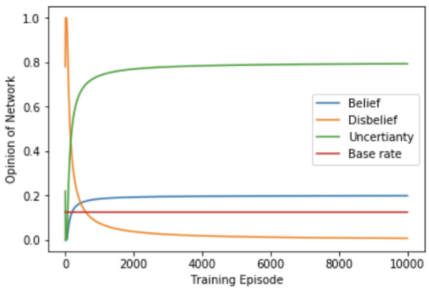

(C) Opinion of NN_1^S in Case III

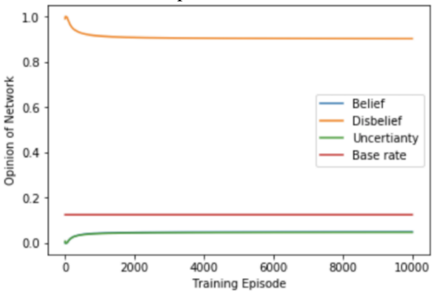

(D) Opinion of NN_1^S in Case IV

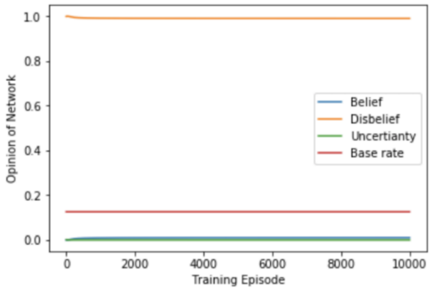

(E) Opinion of NN_1^S in Case V

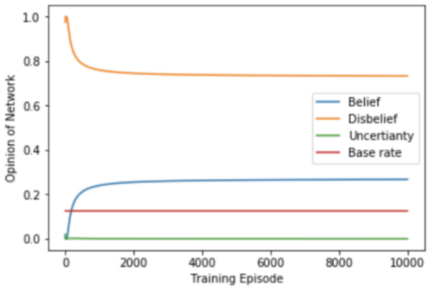

(F) Opinion of NN_1^S in Case VI

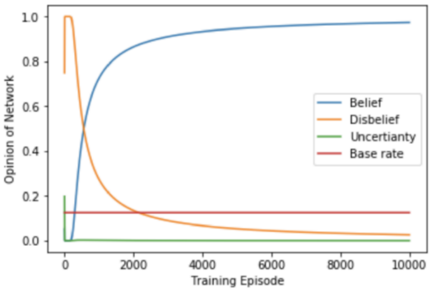

(G) Opinion of NN_2^S in Case I

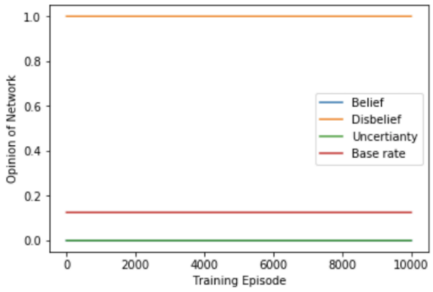

(H) Opinion of NN_2^S in Case II

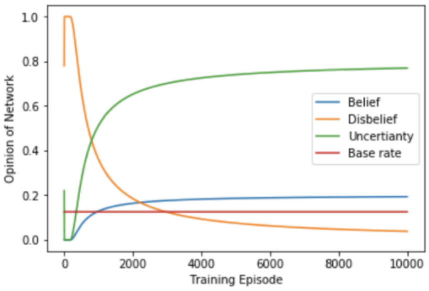

(I) Opinion of NN_2^S in Case III

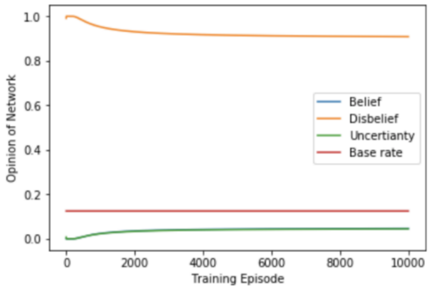

(J) Opinion of NN_2^S in Case IV

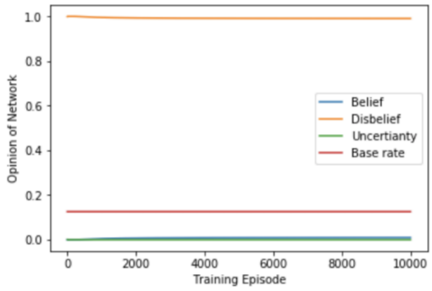

(K) Opinion of NN_2^S in Case V

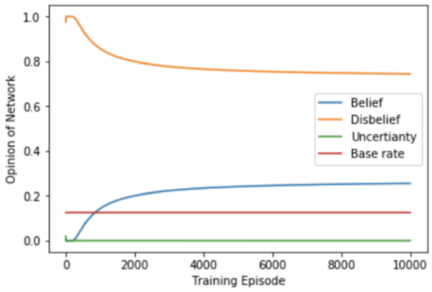

(L) Opinion of NN_2^S in Case VI

Figure S2. Opinion comparison of trained NN_1^S and NN_2^S under six cases stated in Findings. A-F, Opinion of NN_1^S is different when opinion of training data is different. **A,** Belief of NN_1^S settles at 1 and disbelief settles at 0 when opinion of training data is max belief: $(1, 0, 0, 0.5)$. **B,** Disbelief stays at max value 1 when opinion of training data is max disbelief: $(0, 1, 0, 0.5)$. **C,** Belief can be extracted from total uncertainty, and results in higher trust value comparing to **D**. **E,** Belief value is much lower compared to **C**. **F,** Three times more belief mass in dataset's opinion results in a comparable belief value compared to Case III. **G-L,** Opinion of NN_2^S settle at similar value as NN_1^S but with slower speed. The spikes appeared in **A**, **C**, **G**, **I** are caused by high training loss in the early training episodes.

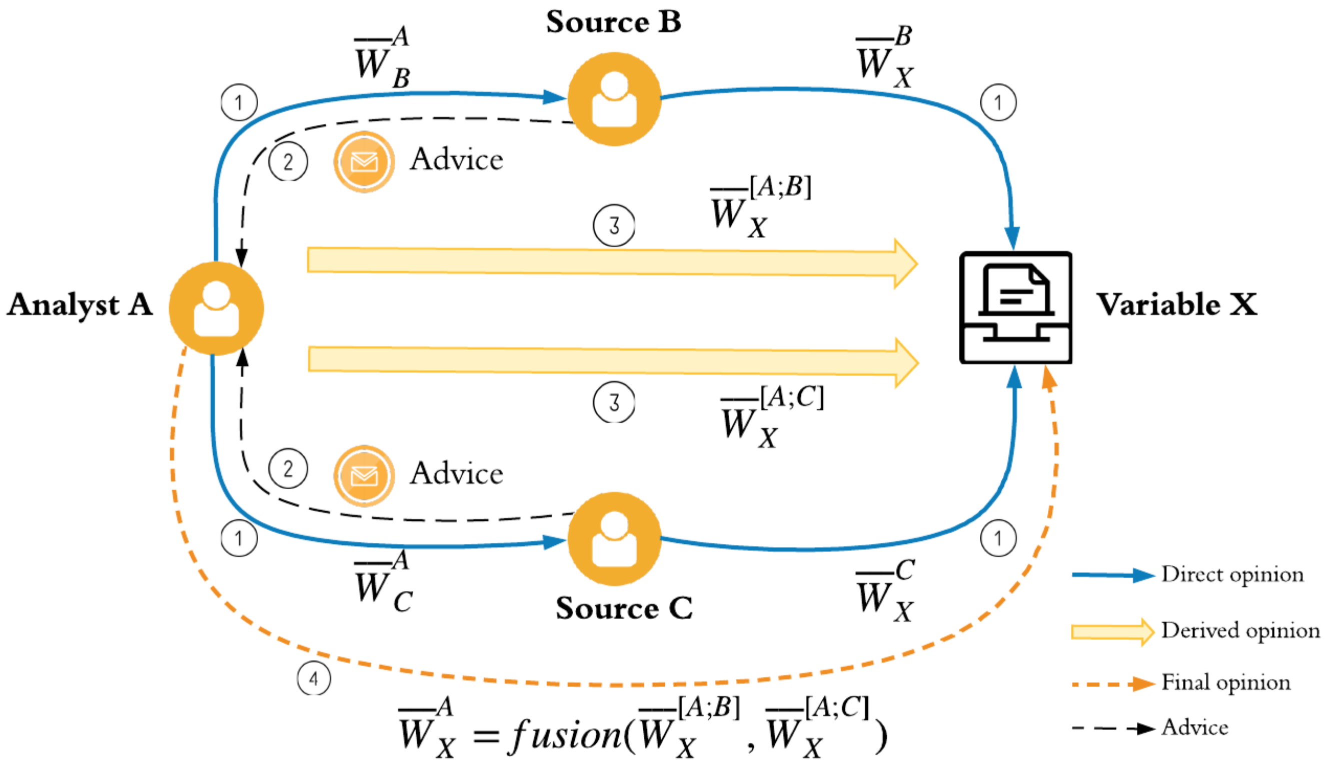

Figure S3. Subjective trust network in social network To derive analyst A's opinion of variable X $\overline{W}_X^A$, (i) the first step of opinion derivation is to evaluate direct opinions such as source B and C's opinions to variable X: $\overline{W}_X^B$, and $\overline{W}_X^C$, and analyst A's opinions to source B and C: $\overline{W}_B^A$, and $\overline{W}_C^A$. (ii) Then sources B and C provide advice to analyst A about variable X, and (iii) analyst A derives opinions of X: $\overline{W}_X^{[A;B]}$, and $\overline{W}_X^{[A;C]}$, by consulting B and C's advice. (iv) Analyst A then combines these opinions and generates the final opinion of variable X: $\overline{W}_X^A$.
